# Targeting GLP-1 Signaling Ameliorates Cystogenesis in a Zebrafish Model of Nephronophthisis

**DOI:** 10.3390/ijms26157366

**Published:** 2025-07-30

**Authors:** Priska Eckert, Maike Nöller, Merle Müller, Rebecca Haas, Johannes Ruf, Henriette Franz, Katharina Moos, Jia-ao Yu, Dongfang Zhao, Wanqiu Xie, Melanie Boerries, Gerd Walz, Toma A. Yakulov

**Affiliations:** 1 Renal Division, University Freiburg Medical Center, Faculty of Medicine, University of Freiburg, 79106 Freiburg, Germanymaike.noeller@uniklinik-freiburg.de (M.N.); merle.mueller@uniklinik-freiburg.de (M.M.); rebecca.haas@uniklinik-freiburg.de (R.H.); jia-ao.yu@uniklinik-freiburg.de (J.-a.Y.); dongfang.zhao@uniklinik-freiburg.de (D.Z.); wanqiu.xie@uniklinik-freiburg.de (W.X.); gerd.walz@uniklinik-freiburg.de (G.W.); 2 Department of Biomedicine, University of Basel, 4001 Basel, Switzerland; henriette.franz@unibas.ch; 3 Institute of Medical Bioinformatics and Systems Medicine (IBSM), Medical Center—University of Freiburg, Faculty of Medicine, University of Freiburg, 79085 Freiburg, Germany; katharina.moos@uniklinik-freiburg.de (K.M.); melanie.boerries@uniklinik-freiburg.de (M.B.); 4 German Cancer Consortium (DKTK), Partner Site University of Freiburg and German Cancer Research Center (DKFZ), 79085 Freiburg, Germany; 5 Signalling Research Centres BIOSS and CIBSS, University of Freiburg, 79085 Freiburg, Germany

**Keywords:** nephronophthisis, GLP-1 signaling, adenosine receptor, cystogenesis, zebrafish, adenylate cyclase

## Abstract

Nephronophthisis (NPH) is the leading genetic cause of end-stage renal disease in children and young adults, but no effective disease-modifying therapies are currently available. Here, we identify glucagon-like peptide-1 (GLP-1) signaling as a novel therapeutic target for NPH through a systematic drug repurposing screen in zebrafish. By simultaneously depleting *nphp1* and *nphp4*, we developed a robust zebrafish model that reproduces key features of human NPH, including glomerular cyst formation. Our screen revealed that dipeptidyl peptidase-4 (DPP4) inhibitors (Omarigliptin and Linagliptin) and GLP-1 receptor agonists (Semaglutide) significantly reduce cystogenesis in a dose-dependent manner. Genetic analysis demonstrated that GLP-1 receptor signaling is important for maintaining pronephros integrity, with *gcgra* and *gcgrb* (GLP-1 receptor genes) playing a particularly important role. Transcriptomic profiling identified adenosine receptor A2ab (*adora2ab*) as a key downstream effector of GLP-1 signaling, which regulates ciliary morphology and prevents cyst formation. Notably, *nphp1/nphp4* double mutant zebrafish exhibited the upregulation of *gcgra* as a compensatory mechanism, which might explain their resistance to cystogenesis. This compensation was disrupted by the targeted depletion of GLP-1 receptors or the inhibition of adenylate cyclase, resulting in enhanced cyst formation, specifically in the mutant background. Our findings establish a signaling cascade from GLP-1 receptors to *adora2ab* in terms of regulating ciliary organization and preventing cystogenesis, offering new therapeutic opportunities for NPH through the repurposing of FDA-approved medications with established safety profiles.

## 1. Introduction

Nephronophthisis (NPH) is the most common genetic cause of end-stage renal disease in children and young adults, but no effective disease-modifying therapies are currently available [1]. Despite decades of research since NPH’s initial description, no disease-modifying therapies currently exist, leaving patients dependent on supportive care until renal replacement therapy becomes necessary [2,3]. The progressive nature of NPH, which is characterized by chronic tubulointerstitial nephritis, corticomedullary cysts, and an inevitable progression to kidney failure, underscores the urgent need for therapeutic interventions [4].

The genetic landscape of NPH encompasses over 25 causative genes (NPHP1–NPHP25), with mutations accounting for approximately 60–70% of cases [5]. The encoded proteins, collectively termed nephrocystins, exhibit remarkable functional convergence at the junctions of primary cilia, centrosomes, and adherens, establishing NPH as a form of ciliopathy [4]. Recent advances in understanding the molecular architecture of nephronophthisis have revealed that these proteins do not function as isolated entities but rather assemble into highly organized, functionally specialized multi-protein complexes that orchestrate ciliary structure and function [6].

The NPHP1-4-8 complex represents the most extensively characterized functional unit, with NPHP1, NPHP4, and NPHP8 (RPGRIP1L) demonstrating strong mutual interactions and biochemical stability [6]. Comprehensive proteomic analyses using localization and affinity purification (LAP)-tagging approaches have shown that NPHP4 serves as the central organizing scaffold within this complex, directly binding both NPHP1 and NPHP8 through distinct interaction domains [7]. The functional importance of this scaffolding role is emphasized by the observation that NPHP4 acts upstream of NPHP1 in a common regulatory pathway, controlling the tyrosine phosphorylation status of NPHP1 and thereby regulating its subcellular localization [8]. NPHP8 (RPGRIP1L) represents a particularly important component, given its dual role in both nephronophthisis and retinal ciliopathies, with mutations not only causing nephronophthisis but also contributing to Joubert syndrome and Leber congenital amaurosis [9]. 

Beyond the core NPHP1-4-8 complex, additional nephrocystin modules include the NPHP5/6 complex at the ciliary transition zone, which plays crucial roles in nucleoporin anchoring and ciliary gating functions [10], and the INVS module comprising INVS (NPHP2), NPHP3, NPHP9, and ANKS6, with INVS serving as the major organizer [11,12]. The hierarchical organization within these complexes has profound implications for understanding disease mechanisms, as the disruption of any component can have cascading effects on the entire module’s function [6]. Recent work has also revealed that nephrocystin-1 loss leads to impaired DNA damage response signaling, the persistent inability to repair DNA lesions, and increased senescence and fibrosis characteristics, representing a paradigm shift from purely ciliary dysfunction models [13]. 

Current therapeutic development for NPH has focused on targeting dysregulated signaling networks, particularly cAMP-mediated pathways. Vasopressin V2 receptor antagonists (tolvaptan and lixivaptan) represent the most extensively studied class, blocking vasopressin-induced adenylyl cyclase activation to reduce the intracellular cAMP levels that drive cyst expansion [14,15,16]. In animal models, OPC31260 lowered renal cAMP levels, inhibited disease development, and caused the regression of established cysts [14]. Somatostatin analogs (octreotide and lanreotide) inhibit multiple cystogenic pathways simultaneously by suppressing secretin-induced cAMP generation, inhibiting vasopressin-induced water permeability, and suppressing cystogenic growth factors like IGF-1 [17,18]. mTOR pathway modulation represents another therapeutic avenue, as mTOR signaling is dysregulated in multiple NPH subtypes and its inhibition can reduce cellular proliferation and cyst growth [19]. Calcium channel modulators and calcimimetics have demonstrated protective effects in preclinical NPH studies by restoring calcium–cAMP balance, which is fundamentally disrupted in ciliopathies [20]. 

Drug repurposing approaches have gained particular attention for NPH, given the challenges of developing therapies for rare diseases. Recent successes include eupatilin, identified through phenotype-based screening, which addresses ciliary transition zone defects caused by CEP290 mutations by stabilizing NPHP5 localization and function [21]. CDK inhibitors, particularly roscovitine, have demonstrated efficacy in targeting dysregulated cell cycle progression, effectively arresting kidney volume expansion and cyst progression in jck/Nek8/Nphp9 mouse models and attenuating renal cyst progression in Cep164-knockout mice [22]. Anti-inflammatory approaches have also shown promise, with methylprednisolone reducing fibrosis and preserving kidney function in rodent models of infantile NPH, while senolytic drugs like FOXO4-DRI effectively eliminated senescent cells and reduced inflammation and tubulointerstitial fibrosis in Glis2-knockout mice [23,24]. Despite these therapeutic advances, the complexity of NPH pathophysiology necessitates the exploration of additional signaling pathways that might offer novel intervention opportunities.

Glucagon-like peptide-1 (GLP-1) is an incretin hormone that plays a central role in glucose homeostasis by enhancing insulin secretion, inhibiting glucagon release, slowing gastric emptying, and promoting satiety [25,26,27]. GLP-1 receptor agonists and dipeptidyl peptidase-4 (DPP4) inhibitors, which prevent the degradation of endogenous GLP-1, are widely used in the treatment of type 2 diabetes [28,29,30]. The primary mechanism of GLP-1 action involves the stimulation of adenylyl cyclase and the elevation of intracellular cAMP levels, a signaling pathway that is fundamentally important for ciliary structure and function [31]. Recent studies have shown that GLP-1 receptor agonists can reduce albuminuria, attenuate renal inflammation, and slow the progression of diabetic kidney disease [32,33,34,35]. However, the potential role of GLP-1 signaling in ciliopathies and cystic kidney diseases remains largely unexplored.

Zebrafish have emerged as a valuable model organism for studying kidney development and disease [36,37]. The zebrafish pronephros, although simpler than the mammalian metanephric kidney, shares important structural and functional similarities, including a blood-filtering glomerulus, pronephric tubules lined with ciliated epithelial cells, and a collecting duct [38,39]. Morpholino-mediated knockdown studies have consistently demonstrated that the depletion of various NPHP genes produces characteristic ciliopathy phenotypes, including pronephric cysts, body curvature defects, hydrocephalus, and situs inversus [40,41,42]. However, recent studies have revealed important discrepancies between morpholino-based knockdown and genetic mutants. While morpholino-mediated depletion consistently produces severe ciliopathy phenotypes, zebrafish with defined nphp mutations show these manifestations with much lower frequency [41]. All nphp1-4-8 mutant zebrafish remain viable and fertile, contrasting with the severe developmental defects seen with morpholino knockdown. The reduced phenotype severity in genetic mutants appears to result from genetic compensation mechanisms, with transcriptome analysis of nphp8 mutant embryos revealing the upregulation of the circadian clock genes cry1a and cry5, which can rescue nephropathy-related phenotypes when overexpressed [41].

Here, we leveraged zebrafish models of NPH to identify and validate novel therapeutic targets. Our findings reveal that GLP-1 receptor agonists and DPP4 inhibitors reduce cystogenesis in zebrafish morphants, implicating adenosine receptor A2ab (adora2ab) and adenylate cyclase-mediated cAMP signaling as critical regulators of pronephric integrity. These results establish GLP-1 signaling as a novel therapeutic target for NPH and identify adenosine receptor signaling as a critical downstream mediator.

## 2. Results

### 2.1. Establishment of a Zebrafish Model for Nephronophthisis and a Drug Screening Platform

**Figure 1:**
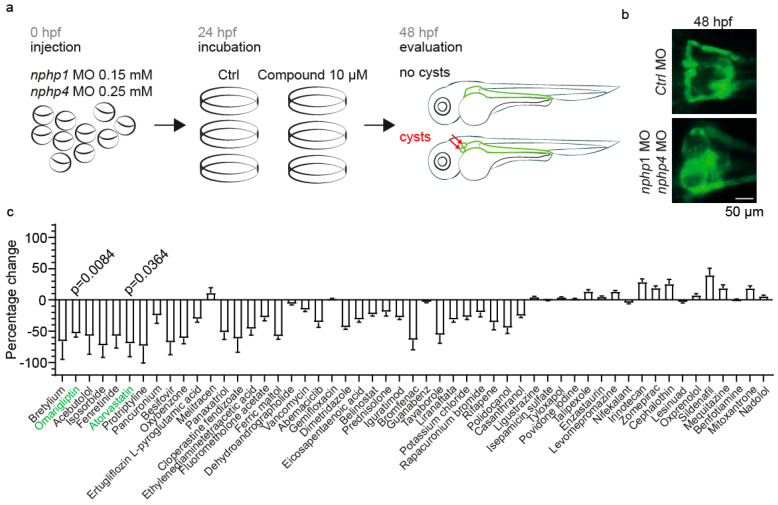
**Drug repurposing screen to identify the compounds affecting cyst formation in a zebrafish nephronophthisis model.** (**a**) Experimental workflow of the drug repurposing screen. Zebrafish oocytes were injected with MO targeting *nphp1* (0.15 mM) and *nphp4* (0.25 mM). At 24 hpf, groups of 20 embryos in triplicate were treated with either 10 µM of test compound or DMSO control. Phenotypic evaluation was performed at 48 hpf, assessing for glomerular cysts (red arrows), a characteristic feature of nephronophthisis. (**b**) Representative fluorescence microscopy images of zebrafish pronephric glomeruli at 48 hpf. The top panel shows normal glomerular morphology in Ctrl MO-injected embryos. Bottom panel demonstrates glomerular cyst formation in *nphp1/nphp4* double-morphant embryos. Scale bar: 50 µm. (**c**) Quantification of the percentage change in cyst formation compared to DMSO controls for all tested compounds. Compounds that were further characterized are highlighted in green (Omarigliptin, Atorvastatin). Data are presented as mean ± SEM.

To identify potential therapeutic compounds for NPH, we developed a zebrafish model by simultaneously depleting *nphp1* and *nphp4* with morpholino oligonucleotides (MO). Zebrafish oocytes of the *Tg(cdh17:GFP;wt1b:GFP)* line were injected with 0.15 mM *nphp1*-MO and 0.25 mM *nphp4*-MO [40], resulting in a robust phenotype characterized by glomerular cyst formation at 48 hpf (Figure 1a,b). These concentrations were chosen because they reproducibly resulted in cyst formation in 40–60% of the embryos at 48 hpf, allowing us to identify those compounds that reduce cystogenesis, as well as those that exacerbate it. This phenotype recapitulates a key pathological feature of NPH, making it a suitable model for drug screening.

We conducted a small-scale drug-repurposing screen with 53 randomly selected FDA-approved drugs (Figure 1c). Groups of 20–25 embryos in triplicate were incubated at 24 hpf in either 10 µM of test compounds or DMSO as a vehicle control, followed by phenotypic evaluation at 48 hpf (Figure 1c). As a positive control, we included Atorvastatin. Atorvastatin has been previously shown to confer renoprotective effects in polycystic kidney disease (PKD) by reducing inflammation and fibrosis [43]. Atorvastatin treatment at concentrations between 2.5 and 25 µM showed no developmental abnormalities in wild-type embryos, but, at 25 µM, it induced cyst formation in approximately 20% of wild-type embryos compared to DMSO controls (Supplementary Figure S1a,b). In *nphp1/nphp4* morphants, Atorvastatin demonstrated a dose-dependent reduction in cyst formation (Supplementary Figure S1c). The expected therapeutic effect of Atorvastatin observed in our screen validates the effectiveness of our zebrafish-based platform for identifying compounds that are relevant to ciliopathies.

### 2.2. Omarigliptin Modulates GLP-1 Signaling and Reduces Cyst Formation in a Zebrafish Nephronophthisis Model

**Figure 2:**
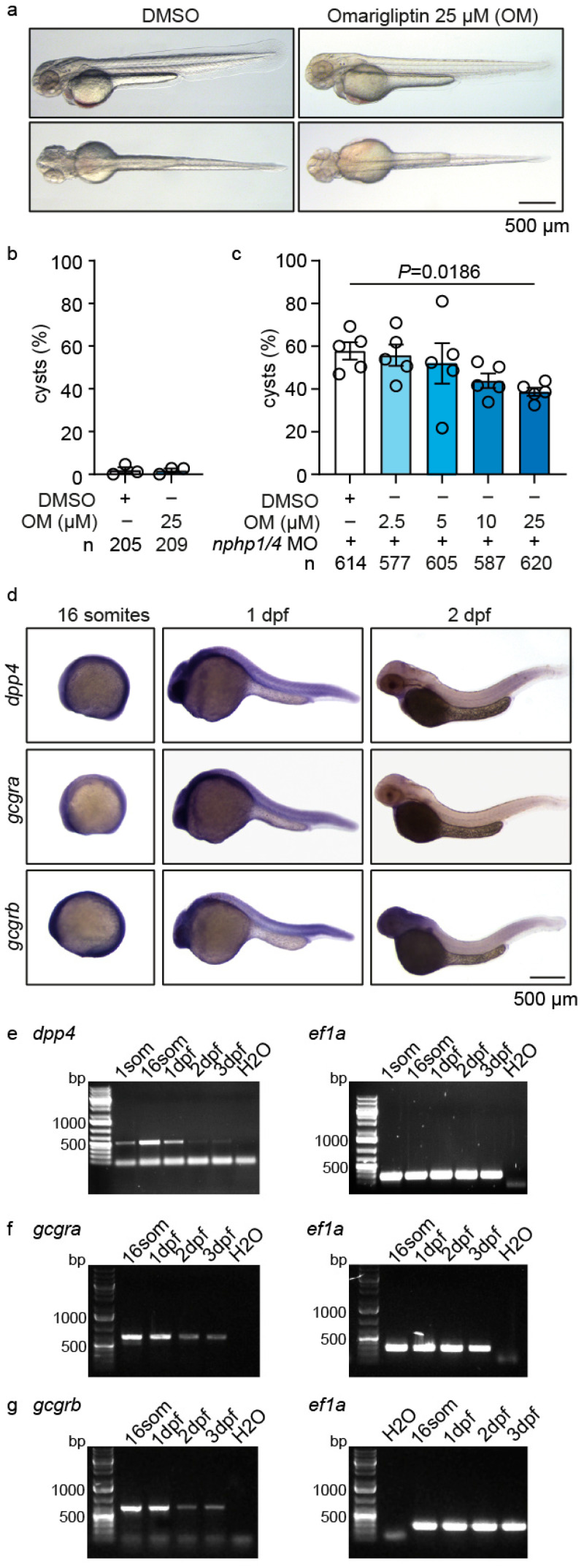
**Omarigliptin treatment reduces cyst formation in a zebrafish nephronophthisis model and expression analysis of target pathway components.** (**a**) Representative brightfield images of wild-type zebrafish embryos at 48 hpf, following 24-h treatment with either DMSO (vehicle control) or Omarigliptin (25 µM), indicating no developmental toxicity. Bar = 500 µm. (**b**) Quantification demonstrating the absence of cyst-inducing effects in wild-type embryos treated with DMSO or 25 µM Omarigliptin. (**c**) The dose-response analysis reveals a concentration-dependent reduction in glomerular cysts in *nphp1/nphp4* double morphant embryos treated with increasing concentrations of Omarigliptin (2.5–25 µM). Statistical comparison between DMSO and 25 µM Omarigliptin treatment shows a significant reduction in cyst formation (*p* = 0.0186). OM, Omarigliptin. (**d**) Whole-mount in situ hybridization showing the temporal and spatial expression patterns of *dpp4*, *gcgra*, and *gcgrb* at the 16-somite stage, 1 dpf, and 2 dpf. Bar = 500 µm. (**e**–**g**) RT-PCR analysis showing the developmental expression profiles of *dpp4* (**e**), *gcgra* (**f**), and *gcgrb* (**g**) from the 16-somite stage to 3 dpf, with *ef1a* serving as the loading control. Data are presented as mean ± SEM. Each circle represents an independent experiment. The total number of embryos analyzed per condition (*n*) is indicated below each graph.

Among the screened compounds, Omarigliptin, a DPP4 inhibitor [44,45,46], emerged as a promising candidate for modulating cystogenesis. To investigate Omarigliptin’s therapeutic potential, we first assessed its safety profile in wild-type embryos. Treatment with Omarigliptin at 25 µM or lower showed no developmental toxicity or morphological abnormalities (Figure 2a). Quantification confirmed that Omarigliptin treatment did not induce cyst formation in wild-type embryos (Figure 2b). In *nphp1/nphp4* morphants, Omarigliptin demonstrated a dose-dependent reduction in glomerular cysts (Figure 2c). 

To understand the molecular basis of Omarigliptin’s therapeutic effect, we examined the expression patterns of key pathway components. In zebrafish, glucagon and GLP-1 signaling operate through a shared set of receptors, *gcgra* and *gcgrb* [47]. Whole-mount in situ hybridization revealed the ubiquitous expression of *dpp4*, *gcgra*, and *gcgrb* throughout the embryo at the 16-somite stage, with expression becoming more restricted by 2 days post-fertilization (dpf), particularly in anterior regions (Figure 2d). Temporal expression analysis by RT-PCR confirmed the sustained expression of *dpp4* from the 16-somite stage through 3 dpf, while both *gcgra* and *gcgrb* showed robust expression at 16 somites and 1 dpf, followed by decreased expression at 2 and 3 dpf (Figure 2e–g).

To further validate the therapeutic potential of DPP4 inhibition, we tested Linagliptin, another DPP4 inhibitor [48,49]. Similar to Omarigliptin, Linagliptin showed no developmental toxicity, and quantification confirmed the absence of cyst formation in Linagliptin-treated controls (Supplementary Figure S2a,b). When applied to *nphp1/nphp4* morphants, Linagliptin demonstrated a concentration-dependent reduction in glomerular cysts (Supplementary Figure S2c). These results suggest a drug class effect rather than a compound-specific phenomenon.

### 2.3. Genetic Analysis Reveals He Role of GLP-1 Receptor Signaling in Cystogenesis

**Figure 3:**
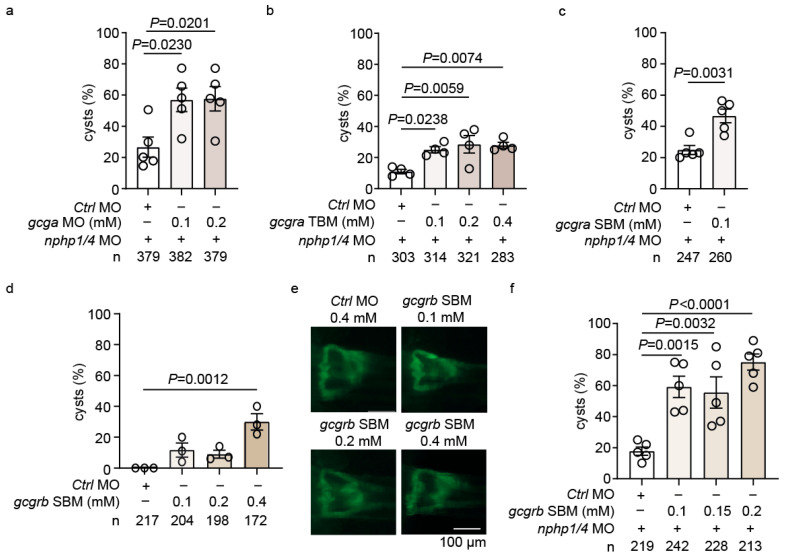
**Modulation of GLP-1/glucagon signaling affects cyst formation in a zebrafish nephronophthisis model**. (**a**) Quantification of cyst formation in embryos injected with suboptimal *nphp1/nphp4* MOs (0.075 mM *nphp1* MO + 0.125 mM *nphp4* MO) co-injected with increasing concentrations of *gcga* MO (0.1–0.2 mM). An increase in cystogenesis is observed compared to embryos injected with control morpholino (Ctrl MO). (**b**) Quantification of cyst formation in embryos injected with suboptimal *nphp1/nphp4* MO co-injected with increasing concentrations of *gcgra* TBM (0.1–0.4 mM). A significant increase in cyst formation is observed compared to Ctrl MO. (**c**) Quantification of cyst formation in embryos injected with suboptimal *nphp1/nphp4* MO co-injected with *gcgra* SBM (0.1 mM), showing a significant increase in cystogenesis compared to Ctrl MO. (**d**) Quantification of cyst formation in wild-type embryos injected with increasing concentrations of *gcgrb* SBM (0.1–0.4 mM). A dose-dependent increase in pronephric glomerular cyst formation is observed compared to Ctrl MO. (**e**) Representative fluorescence microscopy images of pronephric glomeruli at 3 dpf in wild-type embryos injected with either Ctrl MO or increasing concentrations of *gcgrb* SBM (0.1–0.4 mM). Scale bar: 100 µm. (**f**) Quantification of cyst formation in embryos injected with suboptimal *nphp1/nphp4* Mos and co-injected with increasing concentrations of *gcgrb* SBM (0.1–0.2 mM). A significant dose-dependent increase in cystogenesis is observed compared to Ctrl MO. “cysts (%)" on the *y*-axis refers to the percentage of embryos with cysts. Data are presented as mean ± SEM, and the total number of embryos analyzed per condition (*n*) is indicated below each graph.

To investigate the role of GLP-1 signaling in NPH, we performed a systematic genetic analysis of pathway components. The MO-mediated knockdown of *gcga*, which encodes both glucagon and GLP-1 in zebrafish [47], showed no effect on glomerular development in wild-type embryos across all the concentrations tested (0.1–0.4 mM) (Supplementary Figure S3a). However, when co-injected with suboptimal doses of *nphp1/nphp4* MO (0.075 mM *nphp1* MO and 0.125 mM *nphp4* MO, which were half the concentrations used in the initial screen), *gcga* depletion significantly enhanced cyst formation (Figure 3a). This “suboptimal” setup was used throughout the study to partially deplete *nphp1/nphp4*, creating a sensitized background to identify those genetic interactions or compounds that modulate cystogenesis.

We next examined the two zebrafish GLP-1 receptors, *gcgra* and *gcgrb*. Knockdown of *gcgra* using translation-blocking MO (TBM) produced minimal effects in wild-type embryos at concentrations of up to 0.4 mM (Supplementary Figure S3b). However, when combined with suboptimal doses of *nphp1/nphp4* MO, *gcgra*-TBM caused an increase in cyst formation (Figure 3b). Similar results were obtained using a splice-blocking MO (SBM) targeting *gcgra* (Figure 3c, Supplementary Figure S3c).

The depletion of *gcgrb* using SBM demonstrated dose-dependent effects, even in wild-type embryos, with significant cyst formation at higher concentrations (Figure 3d,e). With the *nphp1/nphp4* MO background, *gcgrb* knockdown markedly enhanced cystogenesis (Figure 3f). These findings indicate that *gcgrb* might play a more important role in maintaining pronephros integrity.

To test for functional redundancy between the receptors, we performed combined knockdown of *gcgra* and *gcgrb* at suboptimal concentrations. This epistasis analysis revealed no additive effects on cyst formation, suggesting that both receptors operate in the same pathway to maintain glomerular integrity (Supplementary Figure S3d). 

The specificity of MO-mediated knockdown was confirmed through the validation of splice alterations by RT-PCR and rescue experiments with the co-injection of corresponding mRNAs. The *gcgra*-SBM resulted in the deletion of exon 3, while *gcgrb*-SBM led to the deletion of exon 2, as demonstrated by the presence of shorter PCR products compared to control morpholino-injected embryos (Supplementary Figure S3e,f). Furthermore, the specificity of *gcgrb* knockdown was validated through rescue experiments. The co-injection of *gcgrb* mRNA significantly reduced the cyst formation induced by *gcgrb*-SBM (Supplementary Figure S3g).

### 2.4. Upregulation of GLP-1 Receptor Signaling Mediates Genetic Compensation in Nphp1/Nphp4 Double Mutant Zebrafish

To further investigate the role of GLP-1 signaling in NPH, we generated the *nphp1^ex15-del4^*;*nphp4^sa38686^* homozygous mutant zebrafish line. Surprisingly, the mutants exhibited normal development without apparent phenotypes, despite carrying mutations in both the genes implicated in NPH. Detailed phenotypic analysis confirmed that the *nphp1^ex15-del4^*;*nphp4^sa38686^* mutants showed non-significant cyst formation (Supplementary Figure S4a), displayed normal ciliary morphology in the pronephric ducts (Supplementary Figure S4b), and exhibited normal heart looping without laterality defects (Supplementary Figure S4c,d).

To validate these genetic alterations, we performed RNAseq analysis of the *nphp1^ex15-del4^;nphp4^sa38686^* homozygous mutants and the wild-type siblings. In the *nphp1* locus of the homozygous mutants, a 4-bp deletion was mapped to exon 15, resulting in a frameshift mutation and premature stop codon. In the *nphp4* locus, a splice site mutation was identified, leading to aberrant splicing (Supplementary Figure S5). The absence of NPH-related phenotypes in these double mutants implies the presence of robust genetic compensation mechanisms.

**Figure 4:**
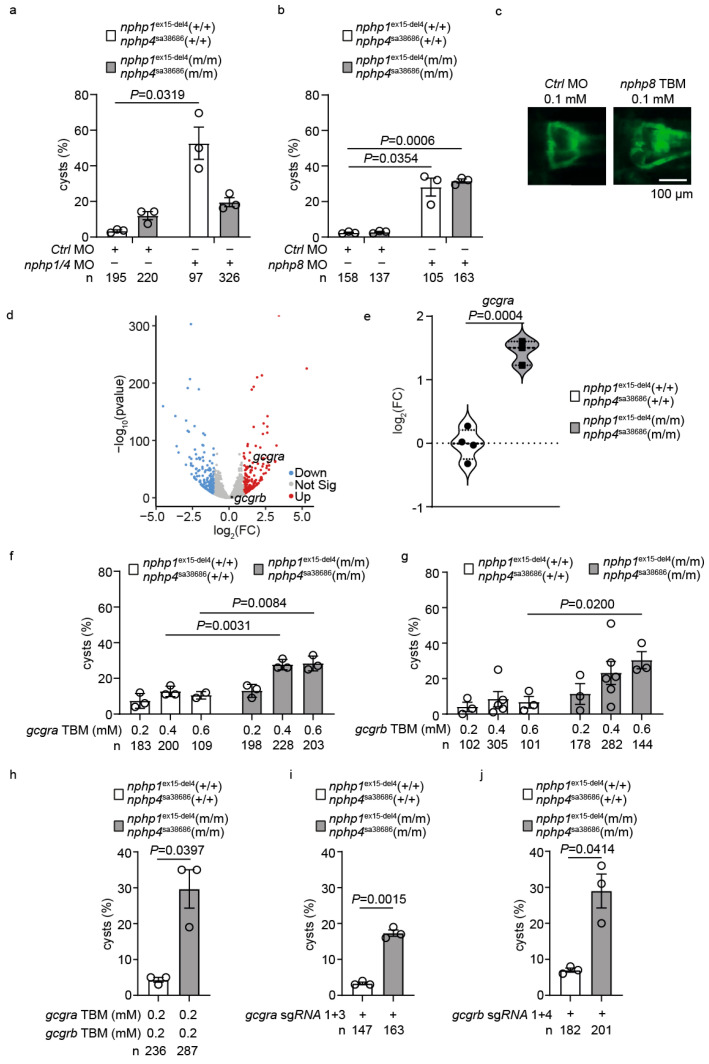
**GLP-1/glucagon receptor depletion enhances cystogenesis in *nphp1^ex15-del4^;nphp4^sa38686^* double mutant zebrafish.** (**a**) Quantification of cyst formation in *nphp1^ex15-del4^;nphp4^sa38686^* homozygous mutant embryos (m/m) and wild-type siblings (+/+) injected with *nphp1/nphp4* MO shows the resistance of mutants to morpholino effects. (**b**) Both *nphp1^ex15-del4^;nphp4^sa38686^* homozygous mutants and wild-type siblings remain sensitive to *nphp8* MO-induced cyst formation. (**c**) Representative fluorescence microscopy images showing normal glomerular morphology in control MO-injected *nphp1^ex15-del4^;nphp4^sa38686^* homozygous mutant embryos and cyst formation following *nphp8* TBM injection. Bar = 100 µm. Data are presented as mean ± SEM. Each circle represents an independent experiment. The total number of embryos analyzed per condition (*n*) is indicated below each graph. (**d**) The volcano plot of RNA sequencing data comparing double mutants to wild-type siblings reveals the significant upregulation of *gcgra* (log_2_FC = 1.5), but not of *gcgrb* (log_2_FC = 0.2). (**e**) Violin plot showing the log_2_FC of *gcgra* expression from RNA sequencing data comparing *nphp1^ex15-del4^;nphp4^sa38686^* homozygous mutants (m/m) to wild-type siblings (+/+). Adj. *p*-value is shown. (**f**) Dose-dependent increase in cyst formation following *gcgra* TBM injection in *nphp1^ex15-del4^;nphp4^sa38686^* homozygous mutant embryos compared to their wild-type siblings. (**g**) Similar dose-dependent enhancement of cystogenesis by *gcgrb* TBM in the *nphp1^ex15-del4^;nphp4^sa38686^* homozygous mutant background. (**h**) The combined knockdown of *gcgra* and *gcgrb* shows enhanced cyst formation, specifically in *nphp1^ex15-del4^;nphp4^sa38686^* homozygous mutant embryos. (**i**,**j**) The sgRNA-mediated targeting of *gcgra* or *gcgrb* leads to increased cyst formation in *nphp1^ex15-del4^;nphp4^sa38686^* homozygous mutant embryos. Bar = 100 µm.

To test this hypothesis, we challenged the double mutants with MO-mediated depletion of *nphp1/nphp4*. While wild-type siblings developed significant glomerular cyst formation (52.3 ± 8.2%, *n* = 97), double mutants showed marked resistance (18.4 ± 3.1%, *n* = 326, *p* = 0.0319) (Figure 4a). To validate the specificity of this resistance, we MO-depleted *nphp8* [50], which induced comparable cystogenesis in both mutants and wild-type siblings (Figure 4b,c). This result confirmed that the resistance was specific to *nphp1/nphp4* depletion and was not a general resistance to cystogenesis.

Principal component analysis (PCA) of the RNAseq data demonstrated a clear separation between mutant and control groups (Supplementary Figure S4e), highlighting distinct transcriptional profiles. Gene ontology enrichment analysis identified a significant upregulation of the pathways associated with metabolic processes, enzyme inhibitor activity, and extracellular matrix regulation in the double mutants (Supplementary Figure S4f). Notably, *gcgra*, encoding one of the GLP-1 receptors, showed significant upregulation (log_2_FC = 1.43, adj *p* = 2.78 × 10^−53^), suggesting enhanced GLP-1 signaling as a potential compensatory mechanism for maintaining kidney integrity despite *nphp1/nphp4* genetic deficiencies (Figure 4d,e, Supplementary Table S1). 

To test whether this upregulation was functionally relevant, we performed targeted genetic perturbations. The injection of *gcgra* TBM induced dose-dependent cystogenesis, specifically in *nphp1^ex15-del4^;nphp4^sa38686^* mutants (Figure 4f). Similarly, *gcgrb* MO depletion enhanced cyst formation in the mutant background (Figure 4g). The combined knockdown of both receptors at suboptimal concentrations resulted in significant cystogenesis in *nphp1^ex15-del4^;nphp4^sa38686^* mutants compared to controls (Figure 4h). CRISPR/Cas9-mediated targeting using paired sgRNAs against *gcgra* or *gcgrb* corroborated these findings, demonstrating enhanced cyst formation, specifically in the double mutants (Figure 4i,j, Supplementary Figure S6). These data establish the upregulation of GLP-1 receptor signaling as an important compensatory mechanism in *nphp1^ex15-del4^;nphp4^sa38686^* mutants.

### 2.5. Transcriptional Profiling Reveals *adora2ab* as a Downstream Effector of GLP-1 Signaling

**Figure 5:**
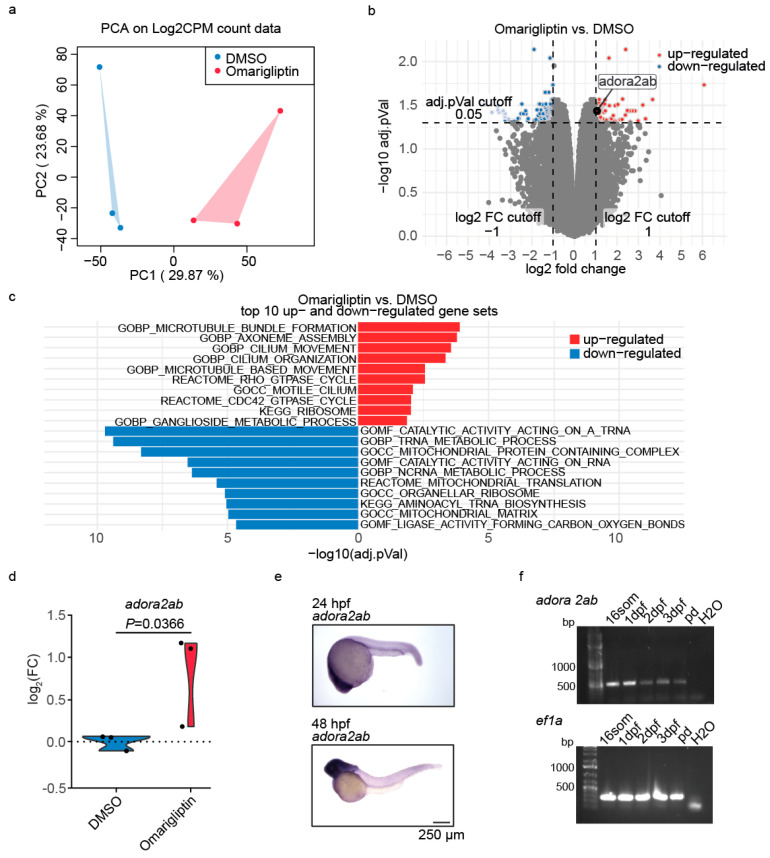
**Transcriptional profiling reveals *adora2ab* regulation in the Omarigliptin-treated nephronophthisis model.** (**a**) Principal component analysis (PCA) of the Log2CPM count data shows the distinct clustering of DMSO- and Omarigliptin-treated *nphp1/nphp4* morphant samples. (**b**) Volcano plot of the RNA sequencing data analysis comparing *nphp1/nphp4* morphant embryos treated with Omarigliptin versus DMSO treatment reveals significant upregulation of *adora2ab*. (**c**) Gene set enrichment analysis identifies cilium-associated processes among the top 10 upregulated pathways (by adjusted *p*-value) in Omarigliptin-treated *nphp1/nphp4* morphant embryos. (**d**) Violin plot showing the log_2_FC of *adora2ab* expression from RNA sequencing data, comparing *nphp1/nphp4* morphant embryos treated with Omarigliptin versus DMSO treatment. Adj. *p*-value is shown. (**e**) Whole-mount in situ hybridization showing spatial expression pattern of *adora2ab* at 24 hpf and 48 hpf. Bar = 250 µm. (**f**) RT-PCR analysis showing the temporal expression profile of *adora2ab* from the 16-somite stage to 3 dpf, with *ef1a* serving as a loading control.

To elucidate the mechanisms underlying Omarigliptin’s therapeutic effects, we performed RNA sequencing analysis comparing *nphp1/nphp4* morphant embryos treated with Omarigliptin versus DMSO-treated controls. PCA and differential gene expression analysis demonstrated a clear distinction between the treated and untreated groups (Figure 5a,b; Supplementary Table S2). Gene set enrichment analysis revealed the significant upregulation of cilium-associated processes, including microtubule bundle formation, cilium movement, and cilium organization (Figure 5c). This enrichment of the ciliary genes suggests that Omarigliptin might act by restoring ciliary function.

Among the significantly upregulated genes, we identified *adora2ab*, encoding adenosine receptor A2A, as a potential downstream effector (Figure 5a,d). Adenosine receptors are G-protein-coupled receptors that signal through cAMP, a second messenger implicated in ciliary function and cystogenesis [51]. Spatial expression analysis by whole-mount in situ hybridization revealed ubiquitous *adora2ab* expression throughout the embryo at 24–48 hpf, with a particularly strong signal in the head region (Figure 5e). Temporal expression analysis by RT-PCR demonstrated sustained *adora2ab* expression from the 16-somite stage, through 3 dpf (Figure 5f).

### 2.6. *adora2ab* Depletion Induces Cystogenesis and Disrupts Ciliary Morphology

**Figure 6:**
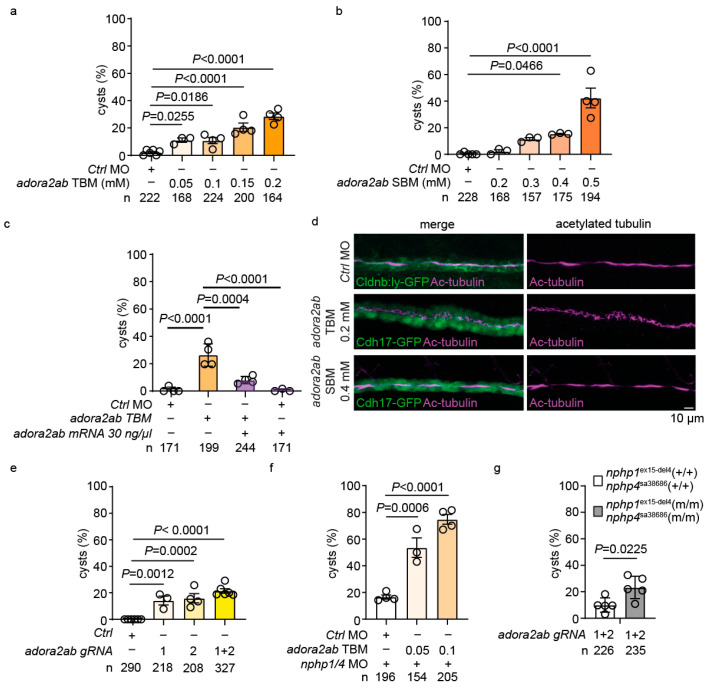
***adora2ab* depletion induces cystogenesis and ciliary defects in zebrafish embryos.** (**a**) Dose-dependent increase in cyst formation following *adora2ab* TBM injection in wild-type embryos. (**b**) Dose-dependent increase in cyst formation following *adora2ab* SBM injection. (**c**) Quantification of cyst formation in rescue experiments. The co-injection of *adora2ab* mRNA (30 ng/μl) significantly reduces cyst formation in *adora2ab* TBM-injected embryos. The control morpholino shows minimal cyst formation. (**d**) Confocal microscopy analysis of pronephric cilia at 48 hpf using acetylated tubulin immunostaining (magenta) in *Tg(wt1b:GFP; cldnb-ly:GFP)* embryos. *adora2ab* TBM injection results in an irregular cilia morphology, while SBM-injected embryos maintain a normal ciliary structure. Bar = 10 µm. (**e**) The sgRNA-mediated targeting of *adora2ab* leads to increased cyst formation in wild-type embryos. (**f**) Enhanced cyst formation in *nphp1/nphp4* morphants co-injected with *adora2ab* TBM. (**g**) Analysis of cyst formation in *nphp1^ex15-del4^;nphp4^sa38686^* homozygous mutants and wild-type siblings following *adora2ab* sgRNA injection (*n* = 226 and 235 embryos). Each circle represents an independent experiment. Data are presented as mean ± SEM.

To investigate the functional role of *adora2ab*, we employed multiple genetic approaches. MO-mediated knockdown using *adora2ab*-TBM [52] induced dose-dependent cyst formation in wild-type embryos (Figure 6a, Supplementary Figure S7a). Similarly, *adora2ab*-SBM [52] demonstrated concentration-dependent effects (Figure 6b, Supplementary Figure S7a). To confirm the specificity of the MO-induced phenotypes, we performed rescue experiments by co-injecting *adora2ab* mRNA with the *adora2ab*-TBM, which significantly attenuated cyst formation (Figure 6c, Supplementary Figure S7b). To examine the cellular basis of these phenotypes, we analyzed ciliary morphology in the pronephric duct using acetylated tubulin immunostaining. *adora2ab*-TBM-injected embryos displayed irregular and disorganized ciliary structures, while SBM-injected embryos maintained relatively normal ciliary morphology (Figure 6d). This might reflect differential effects on maternal versus zygotic *adora2ab* transcripts, as TBM blocks both maternal and zygotic protein synthesis, while SBM affects only zygotic splicing. 

To validate these findings, we employed CRISPR/Cas9-mediated gene targeting. The injection of sgRNAs targeting *adora2ab* (Supplementary Figure S8) resulted in significant cyst formation, with the combined injection of multiple gRNAs showing enhanced effects (Figure 6e). Furthermore, low-dose *adora2ab*-TBM synergistically enhanced cyst formation in *nphp1/nphp4* morphants (Figure 6f). Notably, *adora2ab* targeting using gRNAs induced significantly higher cyst formation in *nphp1^ex15-del4^;nphp4^sa38686^* homozygous mutants compared to the control siblings (Figure 6g).

### 2.7. Functional Validation of the GLP-1-adora2ab Axis in Preventing Cystogenesis

**Figure 7:**
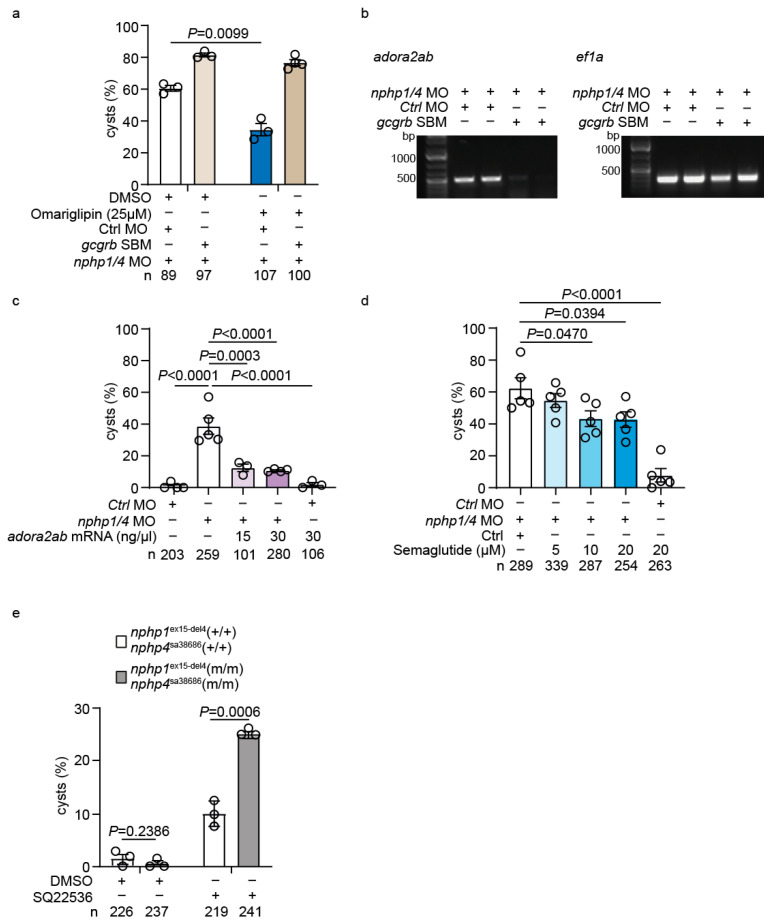
**The *adora2ab* pharmacological modulation of GLP-1 signaling affects cyst formation in a zebrafish nephronophthisis model.** (**a**) Combined treatment with *gcgrb* SBM and Omarigliptin demonstrates that Omarigliptin’s therapeutic effect is abolished in *gcgrb*-depleted nphp1/nphp4 morphant embryos, indicating that Omarigliptin’s cyst-reducing activity requires intact *gcgrb* signaling. (**b**) RT-PCR analysis shows reduced *adora2ab* expression in nphp1/nphp4 morphants co-injected with *gcgrb* SBM, indicating that *adora2ab* acts downstream of *gcgrb* signaling. *ef1a* serves as the loading control. (**c**) Rescue of cyst formation in *nphp1/nphp4* morphant embryos by *adora2ab* mRNA injection. (**d**) Dose-dependent reduction in cyst formation in *nphp1/nphp4* morphant embryos treated with increasing concentrations of Semaglutide, a GLP-1 agonist. (**e**) Quantification of glomerular cyst formation in wild-type siblings (+/+) and homozygous mutants (m/m) treated with the adenylate cyclase inhibitor SQ22536 (100 µM). Wild-type siblings exhibit mild cystogenesis, while homozygous mutants show significantly increased susceptibility, indicating that adenylate cyclase activity is critical for maintaining pronephric integrity in the absence of functional *nphp1/nphp4*. Data are presented as mean ± SEM. Each circle represents an independent experiment. The total number of embryos analyzed per condition (*n*) is indicated below each graph.

To further investigate the therapeutic potential of targeting the GLP-1 pathway, we performed combined genetic and pharmacological interventions. Treatment of *gcgrb*-depleted *nphp1/nphp4* morphant embryos with Omarigliptin failed to reduce cyst formation, indicating that Omarigliptin’s therapeutic effect requires intact *gcgrb* signaling (Figure 7a). RT-PCR analysis showed reduced *adora2ab* expression in *nphp1/nphp4* morphants co-injected with *gcgrb*-SBM, confirming that *adora2ab* acts downstream of *gcgrb* (Figure 7b). 

To validate *adora2ab*’s role in the GLP-1 signaling pathway, we performed rescue experiments with *adora2ab* mRNA injections, which demonstrated a dose-dependent reduction in cyst formation in *nphp1/nphp4* morphant embryos (Figure 7c). This indicates that *adora2ab* upregulation is not merely a correlative finding but functionally relevant for preventing cystogenesis.

Finally, we tested Semaglutide, a GLP-1 receptor agonist, which showed a dose-dependent reduction in cyst formation in *nphp1/nphp4* morphant embryos (Figure 7d). This implies that the direct activation of GLP-1 receptors could reduce cystogenesis.

### 2.8. Adenylate Cyclase (AC) Inhibition Exacerbates Cystogenesis in Nphp1ex15-Del4;Nphp4sa38686 Homozygous Mutants

Given that *adora2ab* is a Gαs-coupled receptor that stimulates AC activity and increases cAMP levels, we hypothesized that cAMP signaling might play a critical role in maintaining pronephric integrity in the absence of functional *nphp1/nphp4*. To investigate the role of AC in cystogenesis, we treated *nphp1^ex15-del4^;nphp4^sa38686^* mutants and their wild-type siblings with the AC inhibitor SQ22536. Control siblings treated with SQ22536 showed only a mild increase in glomerular cyst formation (10.1 ± 1.4%). However, homozygous double mutants exhibited a significantly higher incidence of glomerular cysts (24.9 ± 0.5%) compared to their wild-type siblings (Figure 7e). These findings indicate that *nphp1^ex15-del4^;nphp4^sa38686^* homozygous mutants are particularly susceptible to disruptions in AC activity, supporting the notion that cAMP signaling is important for preventing cystogenesis in this genetic context.

## 3. Discussion

Our study establishes the GLP-1 signaling pathway as a novel therapeutic target in NPH. Through a drug repurposing screen in zebrafish, we identified DPP4 inhibitors (Omarigliptin and Linagliptin) and GLP-1 receptor agonists (Semaglutide) as being effective suppressors of cystogenesis. The dose-dependent reduction in glomerular cysts observed with these compounds suggests a class effect mediated through GLP-1 signaling enhancement. These findings are significant, given these drugs’ established safety profiles and clinical approvals for treating type 2 diabetes.

While GLP-1 signaling has been extensively studied in metabolic disorders, its role in kidney diseases, particularly ciliopathies, remains largely unexplored. GLP-1 receptors are expressed in various kidney cell types, and their activation has shown renoprotective effects in diabetic nephropathy through the reduction of albuminuria, the attenuation of inflammation, and improvement of endothelial function [32,33,34,35]. Our work extends the GLP-1-based therapies’ potential for ciliopathies like NPH.

The identification of *adora2ab* as a downstream effector of GLP-1 signaling provides mechanistic insights into how this pathway maintains glomerular integrity. Adenosine receptors are G protein-coupled receptors that regulate intracellular cAMP levels through their coupling to Gαs or Gαi proteins [51]. Adenosine signaling plays critical roles in renal physiology, particularly in tubular function, glomerular filtration regulation, and responses to ischemic injury [53]. A2A receptors are abundantly expressed in renal tubular epithelial cells and mediate protective responses to acute kidney injury by promoting tubular regeneration and reducing inflammatory responses through cAMP-dependent mechanisms [54]. In the context of cystic kidney diseases, adenosine signaling has emerged as an important regulatory pathway. Studies in PKD models have demonstrated that A2A receptor activation can modulate cyst growth through its effects on cellular proliferation and fluid secretion, with the balance of adenosine receptor subtypes determining whether signaling promotes or inhibits cystogenesis [55]. 

A2a receptors, including *adora2ab*, stimulate adenylate cyclase activity via Gαs proteins, leading to increased cAMP production [52,56]. In ciliopathies, cAMP regulation is particularly complex, as primary cilia both respond to and generate cAMP signals. Disruption of ciliary cAMP homeostasis has been implicated in multiple ciliopathic phenotypes, including cystogenesis, suggesting that *adora2ab* may serve as a nodal point integrating ciliary and metabolic signaling. Recent studies have also highlighted the role of adenosine receptors in ciliary length regulation and ciliary membrane composition, with A2A receptor activation promoting ciliary stability through the PKA-dependent phosphorylation of ciliary proteins [57,58,59]. This pathway may act synergistically with GLP-1 receptor signaling, which also activates adenylate cyclase via Gαs proteins [60,61]. Both pathways converge on cAMP production, which activates downstream effectors such as protein kinase A and exchange proteins directly activated by cAMP (Epac), regulating ion channel activity, cytoskeletal dynamics, and cellular proliferation [62,63,64]. The upregulation of *adora2ab* observed in Omarigliptin-treated embryos suggests that it may act as a mediator of GLP-1 signaling to maintain pronephric integrity by amplifying the cAMP signals necessary for proper ciliary function and tubular homeostasis.

An important technical consideration in our study is the apparent discrepancy between morpholino-based and CRISPR/Cas9-mediated targeting of *adora2ab*. While both approaches primarily affect zygotic gene expression, we observed stronger phenotypic effects with CRISPR/Cas9 compared to splice-blocking morpholinos. This difference most likely reflects the inherent limitations of morpholino-based knockdown approaches, which can show variable efficiency depending on morpholino uptake, stability, and target accessibility. CRISPR/Cas9-mediated targeting creates permanent DNA lesions that result in a more complete loss of function through frameshift mutations and nonsense-mediated decay, whereas morpholinos may allow some residual protein production through incomplete knockdown or alternative splicing mechanisms.

Interestingly, adenosine signaling has been implicated in the regulation of ciliary function and cystogenesis. Studies in PKD models have shown that dysregulated cAMP signaling contributes to cyst growth by promoting cell proliferation and fluid secretion [65,66]. Our findings suggest that *adora2ab* may represent a compensatory mechanism to maintain cAMP homeostasis in the absence of functional *nphp1/nphp4*. However, this compensation appears insufficient under conditions of pharmacological AC inhibition, as evidenced by the increased cyst formation observed in SQ22536-treated double mutants.

The susceptibility of *nphp1^ex15-del4^;nphp4^sa38686^* double mutants to adenylate cyclase inhibition highlights the importance of cAMP signaling as a convergent pathway for both GLP-1 receptor and adenosine receptor signaling. Given that both pathways stimulate adenylate cyclase via Gαs proteins [62,63,64], pharmacological modulation of these pathways may represent a promising therapeutic strategy for NPH. This is consistent with previous studies showing that targeting cAMP production or downstream effectors can ameliorate cyst formation [67,68].

Calcium signaling also plays a critical role in cystogenesis and ciliary function. Calcium-sensitive adenylyl cyclases are regulated by intracellular calcium levels and have been implicated in cyst formation in PKD [66,67]. The interplay between calcium and cAMP signaling is particularly relevant in the context of ciliopathies, as primary cilia serve as calcium-signaling compartments [69,70]. Disruption of calcium homeostasis due to ciliary dysfunction may lead to dysregulated cAMP production and subsequent cyst formation [71,72]. Although Omarigliptin ultimately improves cystogenesis and ciliary architecture, the most parsimonious explanation is that DPP-4 inhibition first augments GLP-1 receptor/Gα_s signaling, thereby increasing cAMP. The ensuing rise in cAMP can modulate Ca^2+^ handling via the PKA- and EPAC-dependent phosphorylation of Ca^2+^ channels, pumps, and IP_3_ receptors [73,74]. We, therefore, propose that Omarigliptin does not directly alter intracellular Ca^2+^ but acts upstream by boosting cAMP, which secondarily re-equilibrates Ca^2+^ homeostasis. 

The genetic compensation observed in *nphp1^ex15-del4^;nphp4^sa38686^* mutants, characterized by the upregulation of *gcgra*, provides insights into the complex genetic interactions underlying NPH pathogenesis. This compensation may explain the absence of phenotypes in these mutants under basal conditions and their resistance to MO-induced cystogenesis. Similar genetic compensation mechanisms have been reported in other zebrafish models of ciliopathies and may involve transcriptional adaptation or functional redundancy among related genes [41,75]. Our study highlights the importance of considering compensatory pathways when developing therapeutic strategies for genetic diseases.

Recent studies have explored the repurposing of small molecules for NPH and related renal ciliopathies [2]. Both knowledge-based repurposing approaches targeting dysregulated signaling pathways and unbiased phenotypic screens have identified promising therapeutic candidates. For instance, eupatilin was found to rescue ciliogenesis defects in CEP290-null cells by stabilizing IQCB1/NPHP5, while Rho-associated protein kinase inhibitors improved RPGRIP1L/NPHP8-associated ciliogenesis defects [21,76]. Our identification of GLP-1-based therapies as potential treatments for NPH adds to this growing list of repurposed compounds with therapeutic potential for ciliopathies. 

Several limitations should be acknowledged. The zebrafish pronephros differs from the human metanephric kidney in complexity, and our morpholino-based model reflects acute gene knockdown rather than chronic genetic defects. Validation in mammalian models and clinical studies will be necessary to establish translational relevance. Future mammalian studies should prioritize several key aspects: (1) the assessment of long-term kidney function parameters, including glomerular filtration rate and proteinuria, in Nphp1/Nphp4 knockout models treated with GLP-1 pathway modulators; (2) the evaluation of renal fibrotic markers such as collagen deposition, α-smooth muscle actin expression, and TGF-β signaling to determine whether GLP-1-based therapies can prevent or reverse established fibrosis; (3) the histopathological analysis of tubular integrity, interstitial inflammation, and glomerular morphology to assess structural preservation; and (4) mechanistic validation of the *adora2ab*-cAMP axis in mammalian kidney cells to confirm pathway conservation across species.

In conclusion, our study identifies the GLP-1 signaling pathway as a promising therapeutic target in NPH, with *adora2ab* as a downstream mediator. These findings offer new avenues for drug repurposing, with established medications that could potentially benefit patients with NPH.

## 4. Materials and Methods

### 4.1. Zebrafish Lines and Maintenance

Zebrafish were maintained according to standard protocols [77] and in compliance with the guidelines of the local animal ethics committee at the Regierungspräsidium Freiburg (protocol code G-21/146 from 07.02.2022). The transgenic lines *Tg(cdh17:GFP;wt1b:GFP)* or *Tg(-8.0 cldnb:Ly-GFP)* were used to visualize pronephric structures [78,79]. To suppress pigmentation, embryos were treated with 0.003% 1-phenyl-2-thiourea (PTU) from 24 hpf. The *nphp1^ex15-del4^* and the *nphp4^sa38686^* mutant lines were previously described [41]. The *nphp1^ex15-del4^*;*nphp4^sa38686^* homozygous mutant zebrafish line in the *Tg(cdh17:GFP;wt1b:GFP)* transgenic background was generated by crossing the single mutants.

### 4.2. Morpholino Knockdown and mRNA Rescue

Antisense morpholino oligonucleotides (MOs) were obtained from Gene Tools. MOs were diluted in 100 mM KCL, 10 mM 4- (2-hydroxyethyl)-1-piperazineethanesulfonic acid and 0.1% phenol red (Sigma-Aldrich). First, 4 nl of this solution was microinjected into fertilized eggs at the one-cell stage. All MOs were co-injected with p53-MO to reduce off-target effects. For rescue experiments, MO-resistant mRNAs were co-injected with the corresponding MO. The following translation-blocking (TBM) and splice-blocking (SBM) MOs were used: 

*nphp1*-TBM (5′-CCCTCTTCTCTTTGGAGGCATGTTG-3′) [40], 

*nphp4*-SBM (5′-ATTTATTCCCCATCCACCTGTGTCA-3′) [40], 

*gcga*-TBM (5′-AGGAAAATACTGGACGCCTTTCATT-3′), 

*nphp8*-SBM (5′-TCTGTCAGTGCAGATTGAGTCACTC-3′) [50], 

*gcgra*-TBM (5′-CAGACAGACGGACATTCCTCATCAC-3′), 

*gcgra*-SBM (5′-AGACACCTGGACACACAAGAGTACA-3′), 

*gcgrb*-TBM (5′-AAACAGAGATGATGTCTGACCTGGA-3′), 

*gcgrb*-SBM (5′-AGACACCTGGACACACAAGAGTACA-3′), 

*adora2ab*-TBM (5′-AAGCAAGCGATGAAGAGGCATCCAT-3′) [52], 

*adora2ab*-SBM (5′-CGGCTGTTCACTCACCTCAATGG-3′) [52], 

standard *control*-MO (5′-CCTCTTACCTCAGTTACAATTTATA-3′), and

*p53*-MO (5′-GCGCCATTGCTTTGCAAGAATTG-3′).

### 4.3. Plasmid Construction and mRNA Synthesis

The coding sequences of *gcgra*, *gcgrb*, and *adora2ab* were amplified from a mix of 1–3 dpf zebrafish cDNA. PCR products were cloned into the pCS2+ vector for in vitro transcription. mRNAs were synthesized using the Message Machine SP6 kit (Ambion). The following primers were used for cloning into a PCS2+ vector with custom restriction sites: 

*gcgrb-for-MluI* (5′-AGAACGCGTATGTCCGTCTGTCTGCTGC-3′), 

*gcgrb-rev-XhoI* (5′-TAGACTCGAGTCAAACGTTGGTTTCCTCCAGC-3′), 

*gcgra-for-MluI* (5′-AGAACGCGTATGTCACAAGTAGTTCTCCTTCTGACTC-3′), 

*gcgra-rev-NotI* (5′-ATGCGGCCGCTCACAAGTTGCTCTCGGCATTG-3′), 

*adora2ab-for-MluI* (5′-AGAACGCGTATGCCTCTTCATCGCTTGCTTT-3′), and

*adora2ab-rev-NotI* (5′-ATGCGGCCGCTCACATTGCGGGCAAAACAATG-3′).

### 4.4. CRISPR/Cas9 Gene Targeting

sgRNAs were designed using http://chopchop.cbu.uib.no/. A PCR-based strategy for the sgRNA template construction was used [80]. sgRNAs were synthesized using the MEGAshortscript Kit (Thermo Fisher Scientific). Purification was performed with the MONARCH RNA Cleanup Kit (New England Biolabs). At this point, 200–300 ng sgRNAs were injected together with 300 ng Cas9 protein at the one-cell stage. For double sgRNA injections, 150 ng of each sgRNA was used. The following sgRNAs were used: 

*gcgra*-sgRNA1 (5′-CGTTTGACCGGTATGCCTGC-3′),

*gcgra*-sgRNA3 (5′-CAGTGAGTGTGGCCTGTCCA-3′),

*gcgrb*-sgRNA1 (5′- GTGCCCAAAGAGTCTCTGCTG-3′), 

gcgrb-sgRNA4 (5′-GGTCTGGTGTACCGCGTGTG-3′),

*adora2ab*-sgRNA1 (5′- GATGTATCGATCCACGGCAA-3′), and

*adora2ab*-sgRNA2 (5′-TGGATACTTTCAGTGGTCAT-3′).

All genetic interventions are summarized in Supplementary Table S3.

### 4.5. Drug Screen and Treatment

For drug screening, embryos were injected with *nphp1* MO (0.15 mM) and *nphp4* MO (0.25 mM) at the one-cell stage. At 24 hpf, embryos were dechorionated and groups of 20–25 embryos in triplicate were incubated with 10 μM of test compounds from an FDA-approved drug library (HY-L022M, MCE) or DMSO as the control. All embryos were additionally incubated in PTU (0.003% 1-phenyl-2-thiourea) to avoid skin pigmentation. At 48 hpf, embryos were evaluated for phenotypes. For dose-response experiments, Omarigliptin, Linagliptin, Atorvastatin, and Semaglutide were tested at concentrations ranging from 2.5 to 25 μM. For adenylate cyclase inhibition, 100 µM SQ22536 (Tocris) was used.

### 4.6. RNA Extraction and RT-PCR

Total RNA was extracted from the embryos using the RNeasy Mini Kit (Qiagen, Hilden, Germany) according to the manufacturer’s instructions. For RNA extraction, 15–50 embryos at the desired stage were collected in an Eppendorf tube, water was removed, and 50μl RNA Protect Tissue Reagent (Qiagen, Hilden, Germany) was added. Embryos were stored at −80°C until RNA extraction. cDNA was synthesized using the ProtoScript First Strand cDNA Synthesis Kit (NEB, Ipswich, MA, USA). Semi-quantitative RT-PCR analysis was performed using gene-specific primers, with *ef1α* as a loading control.

### 4.7. Whole-Mount in Situ Hybridization and Immunohistochemistry

Whole-mount in situ hybridization was performed, as previously described [79]. Briefly, embryos were fixed in 4% PFA, dehydrated in methanol, rehydrated, and hybridized with digoxigenin-labeled antisense RNA probes overnight at 65 °C. After washing, embryos were incubated with anti-digoxigenin-AP antibody (1:5000, Roche) and stained with NBT/BCIP (Roche). Immunohistochemistry was performed according to standard protocols. Briefly, fixed and rehydrated embryos were permeabilized, blocked in PBDT (1× PBS, 1% BSA, 1% DMSO, and 0.1% Triton X-100) with 4% normal goat serum, and incubated with primary mouse monoclonal antibodies against acetylated tubulin (Sigma, 1:100) overnight at 4 °C. After washing, embryos were incubated with secondary antibodies labeled with Cy3 (Jackson ImmunoResearch, West Grove, PA, USA).

### 4.8. RNAseq and Analysis

Total RNA was extracted from 15–50 embryos at 48 hpf using the RNeasy Mini Kit (Qiagen) according to the manufacturer’s protocol. Samples were sent for sequencing to Novogene (Cambridge, UK) on an Illumina platform. RNA sequencing analysis for the *nphp1^ex15-del4^;nphp4^sa38686^* double mutants was performed on the Galaxy server according to the protocol described by Batut et al. [81]. The fastq. files were aligned with the zebrafish reference genome GRCz11. For the Omarigliptin versus a DMSO comparison, raw RNA sequencing reads were quality-controlled with FastQC (v0.11.8) and pre-processed with cutadapt (v4.0) to trim the low-quality bases, adapter sequences, and remove low-quality reads. Processed reads were aligned against the zebrafish reference genome GRCz11 from Ensembl (v110) using STAR (v2.6.1e). Gene counts were obtained during alignment using the “--quantMode GeneCounts” option. Gene count normalization and differential gene expression analysis were performed with the R package limma (v3.52.4). Gene set enrichment analysis was carried out using the R package gage (v2.46.1), based on gene sets retrieved with the R package msigdbr (v7.5.1).

### 4.9. Microscopy and Imaging

Embryos were analyzed at 48–55 hpf under a Leica M205 FA epifluorescent microscope. Images were obtained with a Leica DFC450C camera and processed with the Leica Application Suite (Leica Microsystems, Wetzlar, Germany). For confocal imaging, a Zeiss LSM 510 DUO confocal microscope was used with a LD LCI Plan-Apochromat 25×/0.8 objective (Carl Zeiss, Oberkochen, Germany).

### 4.10. Statistical Analysis

Statistical significance for multiple comparisons was determined using Dunnett’s test, and for single comparisons using Student’s *t*-test (*p* < 0.05) in GraphPad Prism 10 (Boston, MA, USA).

ARRIVE compliance documentation is available in the Supplementary Materials.

## Data Availability

RNAseq data are available at GEO, accession numbers GSE291847 and GSE291774.

## Supplementary Materials

The following supporting information can be downloaded at: https://www.mdpi.com/article/10.3390/ijms26157366/s1.

## Abbreviations

The following abbreviations are used in this manuscript:

hpf	Hours post-fertilization
NPH	Nephronophthisis
GLP-1	Glucagon-like peptide-1
DPP4	Dipeptidyl peptidase-4
dpf	Days post-fertilization
SBM	Splice-blocking morpholino oligonucleotide
TBM	Translation-blocking morpholino oligonucleotide
MO	Morpholino oligonucleotide
AC	Adenylate cyclase
PCA	Principal component analysis

## References

[1] Hildebrandt F., Benzing T., Katsanis N. (2011). Ciliopathies. N. Engl. J. Med..

[2] Benmerah A., Briseño-Roa L., Annereau J.-P., Saunier S. (2023). Repurposing Small Molecules for Nephronophthisis and Related Renal Ciliopathies. Kidney Int..

[3] Stokman M.F., Saunier S., Benmerah A. (2021). Renal Ciliopathies: Sorting Out Therapeutic Approaches for Nephronophthisis. Front. Cell Dev. Biol..

[4] Wolf M.T.F., Bonsib S.M., Larsen C.P., Hildebrandt F. (2024). Nephronophthisis: A Pathological and Genetic Perspective. Pediatr. Nephrol..

[5] Gupta S., Ozimek-Kulik J.E., Phillips J.K. (2021). Nephronophthisis-Pathobiology and Molecular Pathogenesis of a Rare Kidney Genetic Disease. Genes.

[6] Sang L., Miller J.J., Corbit K.C., Giles R.H., Brauer M.J., Otto E.A., Baye L.M., Wen X., Scales S.J., Kwong M. (2011). Mapping the Nephronophthisis-Joubert-Meckel-Gruber Protein Network Reveals Ciliopathy Disease Genes and Pathways. Cell.

[7] Mollet G., Silbermann F., Delous M., Salomon R., Antignac C., Saunier S. (2005). Characterization of the Nephrocystin/Nephrocystin-4 Complex and Subcellular Localization of Nephrocystin-4 to Primary Cilia and Centrosomes. Hum. Mol. Genet..

[8] Benzing T., Gerke P., Höpker K., Hildebrandt F., Kim E., Walz G. (2001). Nephrocystin Interacts with Pyk2, p130Cas, and Tensin and Triggers Phosphorylation of Pyk2. Proc. Natl. Acad. Sci. USA.

[9] Arts H.H., Doherty D., van Beersum S.E.C., Parisi M.A., Letteboer S.J.F., Gorden N.T., Peters T.A., Märker T., Voesenek K., Kartono A. (2007). Mutations in the Gene Encoding the Basal Body Protein RPGRIP1L, a Nephrocystin-4 Interactor, Cause Joubert Syndrome. Nat. Genet..

[10] Schäfer T., Pütz M., Lienkamp S., Ganner A., Bergbreiter A., Ramachandran H., Gieloff V., Gerner M., Mattonet C., Czarnecki P.G. (2008). Genetic and Physical Interaction between the NPHP5 and NPHP6 Gene Products. Hum. Mol. Genet..

[11] Srivastava S., Molinari E., Raman S., Sayer J.A. (2018). Many Genes—One Disease? Genetics of Nephronophthisis (NPHP) and NPHP-Associated Disorders. Front. Pediatr..

[12] Hoff S., Halbritter J., Epting D., Frank V., Nguyen T.-M.T., van Reeuwijk J., Boehlke C., Schell C., Yasunaga T., Helmstädter M. (2013). ANKS6 Is a Central Component of a Nephronophthisis Module Linking NEK8 to INVS and NPHP3. Nat. Genet..

[13] Garví E.S., Biermans S., Knoers N.V.A.M., van Eerde A.M., Masereeuw R., Slaats G.G., van Genderen A.M., Janssen M.J. (2025). Loss of Nephronophthisis-Associated Nephrocystin-1 Impairs DNA Damage Repair in Kidney Organoids. bioRxiv.

[14] Gattone V.H., Wang X., Harris P.C., Torres V.E. (2003). Inhibition of Renal Cystic Disease Development and Progression by a Vasopressin V2 Receptor Antagonist. Nat. Med..

[15] Wang X., Constans M.M., Chebib F.T., Torres V.E., Pellegrini L. (2019). Effect of a Vasopressin V2 Receptor Antagonist on Polycystic Kidney Disease Development in a Rat Model. Am. J. Nephrol..

[16] Strong A., Muneeruddin S., Parrish R., Lui D., Conley S.B. (2018). Isosorbide Dinitrate in Nephronophthisis Treatment. Am. J. Med. Genet. A.

[17] Hogan M.C., Masyuk T.V., Page L.J., Kubly V.J., Bergstralh E.J., Li X., Kim B., King B.F., Glockner J., Holmes D.R. (2010). Randomized Clinical Trial of Long-Acting Somatostatin for Autosomal Dominant Polycystic Kidney and Liver Disease. J. Am. Soc. Nephrol..

[18] Ghosh A.K., Hurd T., Hildebrandt F. (2012). 3D Spheroid Defects in NPHP Knockdown Cells Are Rescued by the Somatostatin Receptor Agonist Octreotide. Am. J. Physiol. Ren. Physiol..

[19] Gattone V.H., Sinders R.M., Hornberger T.A., Robling A.G. (2009). Late Progression of Renal Pathology and Cyst Enlargement Is Reduced by Rapamycin in a Mouse Model of Nephronophthisis. Kidney Int..

[20] Chen N.X., Moe S.M., Eggleston-Gulyas T., Chen X., Hoffmeyer W.D., Bacallao R.L., Herbert B.S., Gattone V.H. (2011). Calcimimetics Inhibit Renal Pathology in Rodent Nephronophthisis. Kidney Int..

[21] Kim Y.J., Kim S., Jung Y., Jung E., Kwon H.J., Kim J. (2018). Eupatilin Rescues Ciliary Transition Zone Defects to Ameliorate Ciliopathy-Related Phenotypes. J. Clin. Investig..

[22] Airik R., Airik M., Schueler M., Bates C.M., Hildebrandt F. (2019). Roscovitine Blocks Collecting Duct Cyst Growth in Cep164-Deficient Kidneys. Kidney Int..

[23] Jin H., Zhang Y., Ding Q., Wang S.S., Rastogi P., Dai D.-F., Lu D., Purvis M., Cao C., Wang A. (2019). Epithelial Innate Immunity Mediates Tubular Cell Senescence after Kidney Injury. JCI Insight.

[24] Jin H., Zhang Y., Liu D., Wang S.S., Ding Q., Rastogi P., Purvis M., Wang A., Elhadi S., Ren C. (2020). Innate Immune Signaling Contributes to Tubular Cell Senescence in the Glis2 Knockout Mouse Model of Nephronophthisis. Am. J. Pathol..

[25] Campbell J.E., Drucker D.J. (2013). Pharmacology, Physiology, and Mechanisms of Incretin Hormone Action. Cell Metab..

[26] van Bloemendaal L., ten Kulve J.S., la Fleur S.E., Ijzerman R.G., Diamant M. (2014). Effects of Glucagon-like Peptide 1 on Appetite and Body Weight: Focus on the CNS. J. Endocrinol..

[27] Nauck M.A., Meier J.J. (2016). The Incretin Effect in Healthy Individuals and Those with Type 2 Diabetes: Physiology, Pathophysiology, and Response to Therapeutic Interventions. Lancet Diabetes Endocrinol..

[28] Nauck M.A., Meier J.J., Cavender M.A., Abd El Aziz M., Drucker D.J. (2017). Cardiovascular Actions and Clinical Outcomes With Glucagon-Like Peptide-1 Receptor Agonists and Dipeptidyl Peptidase-4 Inhibitors. Circulation.

[29] Andersen A., Lund A., Knop F.K., Vilsbøll T. (2018). Glucagon-like Peptide 1 in Health and Disease. Nat. Rev. Endocrinol..

[30] Deacon C.F. (2018). Peptide Degradation and the Role of DPP-4 Inhibitors in the Treatment of Type 2 Diabetes. Peptides.

[31] Zheng Z., Zong Y., Ma Y., Tian Y., Pang Y., Zhang C., Gao J. (2024). Glucagon-like Peptide-1 Receptor: Mechanisms and Advances in Therapy. Sig Transduct. Target. Ther..

[32] Muskiet M.H.A., Smits M.M., Morsink L.M., Diamant M. (2014). The Gut-Renal Axis: Do Incretin-Based Agents Confer Renoprotection in Diabetes?. Nat. Rev. Nephrol..

[33] Kawanami D., Takashi Y. (2020). GLP-1 Receptor Agonists in Diabetic Kidney Disease: From Clinical Outcomes to Mechanisms. Front. Pharmacol..

[34] Greco E.V., Russo G., Giandalia A., Viazzi F., Pontremoli R., De Cosmo S. (2019). GLP-1 Receptor Agonists and Kidney Protection. Medicina.

[35] Lee B., Holstein-Rathlou N.-H., Sosnovtseva O., Sørensen C.M. (2023). Renoprotective Effects of GLP-1 Receptor Agonists and SGLT-2 Inhibitors—Is Hemodynamics the Key Point?. Am. J. Physiol.-Cell Physiol..

[36] Drummond I.A. (2005). Kidney Development and Disease in the Zebrafish. J. Am. Soc. Nephrol..

[37] Morales E.E., Wingert R.A., Miller R.K. (2017). Zebrafish as a Model of Kidney Disease. Kidney Development and Disease.

[38] Drummond I. (2003). Making a Zebrafish Kidney: A Tale of Two Tubes. Trends Cell Biol..

[39] Wingert R.A., Selleck R., Yu J., Song H.-D., Chen Z., Song A., Zhou Y., Thisse B., Thisse C., McMahon A.P. (2007). The Cdx Genes and Retinoic Acid Control the Positioning and Segmentation of the Zebrafish Pronephros. PLoS Genet..

[40] Slanchev K., Pütz M., Schmitt A., Kramer-Zucker A., Walz G. (2011). Nephrocystin-4 Is Required for Pronephric Duct-Dependent Cloaca Formation in Zebrafish. Hum. Mol. Genet..

[41] Kayser N., Zaiser F., Veenstra A.C., Wang H., Göcmen B., Eckert P., Franz H., Köttgen A., Walz G., Yakulov T.A. (2022). Clock Genes Rescue Nphp Mutations in Zebrafish. Hum. Mol. Genet..

[42] Zhou W., Dai J., Attanasio M., Hildebrandt F. (2010). Nephrocystin-3 Is Required for Ciliary Function in Zebrafish Embryos. Am. J. Physiol.-Ren. Physiol..

[43] Ecder T. (2016). Statins in the Treatment of Autosomal Dominant Polycystic Kidney Disease. Nephrol. Dial. Transplant..

[44] Hattori S. (2020). Omarigliptin Decreases Inflammation and Insulin Resistance in a Pleiotropic Manner in Patients with Type 2 Diabetes. Diabetol. Metab. Syndr..

[45] Krishna R., Addy C., Tatosian D., Glasgow X.S., Gendrano Iii I.N., Robberechts M., Haazen W., de Hoon J.N., Depré M., Martucci A. (2016). Pharmacokinetics and Pharmacodynamics of Omarigliptin, a Once-Weekly Dipeptidyl Peptidase-4 (DPP-4) Inhibitor, After Single and Multiple Doses in Healthy Subjects. J. Clin. Pharmacol..

[46] Tan X. (2016). Omarigliptin for the Treatment of Type 2 Diabetes. Endocrine.

[47] Mojsov S. (2000). Glucagon-like Peptide-1 (GLP-1) and the Control of Glucose Metabolism in Mammals and Teleost Fish1. Am. Zool..

[48] Jelsing J., Vrang N., van Witteloostuijn S.B., Mark M., Klein T. (2012). The DPP4 Inhibitor Linagliptin Delays the Onset of Diabetes and Preserves β-Cell Mass in Non-Obese Diabetic Mice. J. Endocrinol..

[49] Mikov M., Pavlović N., Stanimirov B., Đanić M., Goločorbin-Kon S., Stankov K., Al-Salami H. (2020). DPP-4 Inhibitors: Renoprotective Potential and Pharmacokinetics in Type 2 Diabetes Mellitus Patients with Renal Impairment. Eur. J. Drug Metab. Pharmacokinet..

[50] Khanna H., Davis E.E., Murga-Zamalloa C.A., Estrada-Cuzcano A., Lopez I., den Hollander A.I., Zonneveld M.N., Othman M.I., Waseem N., Chakarova C.F. (2009). A Common Allele in RPGRIP1L Is a Modifier of Retinal Degeneration in Ciliopathies. Nat. Genet..

[51] Fredholm B.B., Ijzerman A.P., Jacobson K.A., Klotz K.-N., Linden J. (2001). International Union of Pharmacology. XXV. Nomenclature and Classification of Adenosine Receptors. Pharmacol. Rev..

[52] Gessler S., Guthmann C., Schuler V., Lilienkamp M., Walz G., Yakulov T.A. (2022). Control of Directed Cell Migration after Tubular Cell Injury by Nucleotide Signaling. Int. J. Mol. Sci..

[53] Day Y.-J., Huang L., McDuffie M.J., Rosin D.L., Ye H., Chen J.-F., Schwarzschild M.A., Fink J.S., Linden J., Okusa M.D. (2003). Renal Protection from Ischemia Mediated by A2A Adenosine Receptors on Bone Marrow-Derived Cells. J. Clin. Investig..

[54] Grenz A., Osswald H., Eckle T., Yang D., Zhang H., Tran Z.V., Klingel K., Ravid K., Eltzschig H.K. (2008). The Reno-Vascular A2B Adenosine Receptor Protects the Kidney from Ischemia. PLoS Med..

[55] Pastor-Anglada M., Pérez-Torras S. (2018). Emerging Roles of Nucleoside Transporters. Front. Pharmacol..

[56] Vincent I.S., Okusa M.D. (2015). Adenosine 2A Receptors in Acute Kidney Injury. Acta Physiol..

[57] Hilgendorf K.I., Johnson C.T., Jackson P.K. (2016). The Primary Cilium as a Cellular Receiver: Organizing Ciliary GPCR Signaling. Curr. Opin. Cell Biol..

[58] Grenz A., Homann D., Eltzschig H.K. (2011). Extracellular Adenosine: A Safety Signal That Dampens Hypoxia-Induced Inflammation During Ischemia. Antioxid. Redox Signal.

[59] Wheway G., Nazlamova L., Hancock J.T. (2018). Signaling through the Primary Cilium. Front. Cell Dev. Biol..

[60] Sonoda N., Imamura T., Yoshizaki T., Babendure J.L., Lu J.-C., Olefsky J.M. (2008). β-Arrestin-1 Mediates Glucagon-like Peptide-1 Signaling to Insulin Secretion in Cultured Pancreatic β Cells. Proc. Natl. Acad. Sci. USA.

[61] Das A., Geetha K.M., Hazarika I. (2020). Contemporary Updates on the Physiology of Glucagon like Peptide-1 and Its Agonist to Treat Type 2 Diabetes Mellitus. Int. J. Pept. Res. Ther..

[62] Leech C.A., Dzhura I., Chepurny O.G., Kang G., Schwede F., Genieser H.-G., Holz G.G. (2011). Molecular Physiology of Glucagon-like Peptide-1 Insulin Secretagogue Action in Pancreatic β Cells. Prog. Biophys. Mol. Biol..

[63] Takeda Y., Amano A., Noma A., Nakamura Y., Fujimoto S., Inagaki N. (2011). Systems Analysis of GLP-1 Receptor Signaling in Pancreatic β-Cells. Am. J. Physiol.-Cell Physiol..

[64] Zhang X., Cao C., Zheng F., Liu C., Tian X. (2025). Therapeutic Potential of GLP-1 Receptor Agonists in Diabetes and Cardiovascular Disease: Mechanisms and Clinical Implications. Cardiovasc. Drugs Ther..

[65] Torres V.E., Harris P.C., Pirson Y. (2007). Autosomal Dominant Polycystic Kidney Disease. Lancet.

[66] Rees S., Kittikulsuth W., Roos K., Strait K.A., Van Hoek A., Kohan D.E. (2014). Adenylyl Cyclase 6 Deficiency Ameliorates Polycystic Kidney Disease. J. Am. Soc. Nephrol..

[67] Sussman C.R., Wang X., Chebib F.T., Torres V.E. (2020). Modulation of Polycystic Kidney Disease by G-Protein Coupled Receptors and Cyclic AMP Signaling. Cell. Signal..

[68] Chebib F.T., Sussman C.R., Wang X., Harris P.C., Torres V.E. (2015). Vasopressin and Disruption of Calcium Signalling in Polycystic Kidney Disease. Nat. Rev. Nephrol..

[69] Jin X., Mohieldin A.M., Muntean B.S., Green J.A., Shah J.V., Mykytyn K., Nauli S.M. (2014). Cilioplasm Is a Cellular Compartment for Calcium Signaling in Response to Mechanical and Chemical Stimuli. Cell. Mol. Life Sci..

[70] Moore B.S., Stepanchick A.N., Tewson P.H., Hartle C.M., Zhang J., Quinn A.M., Hughes T.E., Mirshahi T. (2016). Cilia Have High cAMP Levels That Are Inhibited by Sonic Hedgehog-Regulated Calcium Dynamics. Proc. Natl. Acad. Sci. USA.

[71] Brill A.L., Fischer T.T., Walters J.M., Marlier A., Sewanan L.R., Wilson P.C., Johnson E.K., Moeckel G., Cantley L.G., Campbell S.G. (2020). Polycystin 2 Is Increased in Disease to Protect against Stress-Induced Cell Death. Sci. Rep..

[72] Kleene S.J. (2022). Regenerative Calcium Currents in Renal Primary Cilia. Front. Physiol..

[73] Vajanaphanich M., Schultz C., Tsien R.Y., Traynor-Kaplan A.E., Pandol S.J., Barrett K.E. (1995). Cross-Talk between Calcium and cAMP-Dependent Intracellular Signaling Pathways. Implications for Synergistic Secretion in T84 Colonic Epithelial Cells and Rat Pancreatic Acinar Cells. J. Clin. Investig..

[74] Zaccolo M., Pozzan T. (2003). CAMP and Ca2+ Interplay: A Matter of Oscillation Patterns. Trends Neurosci..

[75] El-Brolosy M.A., Stainier D.Y.R. (2017). Genetic Compensation: A Phenomenon in Search of Mechanisms. PLoS Genet..

[76] Lake A.V.R., Smith C.E.L., Natarajan S., Basu B., Best S.K., Stevenson T., Trowbridge R., Grellscheid S.N., Bond J., Foster R. (2025). Drug and siRNA Screens Identify ROCK2 as a Therapeutic Target for Ciliopathies. Commun. Med..

[77] Westerfield M. (1995). The Zebrafish Book. A Guide for the Laboratory Use of Zebrafish (Danio Rerio).

[78] Yakulov T.A., Todkar A.P., Slanchev K., Wiegel J., Bona A., Groß M., Scholz A., Hess I., Wurditsch A., Grahammer F. (2018). CXCL12 and MYC Control Energy Metabolism to Support Adaptive Responses after Kidney Injury. Nat. Commun..

[79] Schoels M., Zhuang M., Fahrner A., Küchlin S., Sagar, Franz H., Schmitt A., Walz G., Yakulov T.A. (2021). Single-Cell mRNA Profiling Reveals Changes in Solute Carrier Expression and Suggests a Metabolic Switch during Zebrafish Pronephros Development. Am. J. Physiol.-Ren. Physiol..

[80] Hu Z., Wang L., Shi Z., Jiang J., Li X., Chen Y., Li K., Luo D. (2019). Customized One-Step Preparation of sgRNA Transcription Templates via Overlapping PCR Using Short Primers and Its Application in Vitro and in Vivo Gene Editing. Cell Biosci..

[81] Batut B., van den Beek M., Doyle M.A., Soranzo N. (2021). RNA-Seq Data Analysis in Galaxy. Methods Mol. Biol..

