# Supplementary material for: Targeting GLP-1 Signaling Ameliorates Cystogenesis in a Zebrafish Model of Nephronophthisis
Source: Int J Mol Sci. 2025 Jul 30;26(15):7366. doi: 10.3390/ijms26157366 (PMC12347336; doi:10.3390/ijms26157366)
Supplement: Supplementary file 1 [file ijms-26-07366-s001.zip › Supplementary Figures.pdf]

# SUPPLEMENTARY FIGURES

## Targeting GLP-1 signaling ameliorates cystogenesis in a zebrafish model of nephronophthisis

Priska Eckert[1§], Maike Nöller[1§], Merle Müller[1§], Rebecca Haas[1§], Johannes Ruf[1§], Henriette Franz[2], Katharina Moos[3], Jia-ao Yu[1], Dongfang Zhao[1], Wanqiu Xie[1], Melanie Boerries[3,4], Gerd Walz[1,5], Toma A. Yakulov[1,*]

[1] Renal Division, University Freiburg Medical Center, Faculty of Medicine, University of Freiburg, Germany

[2] Department of Biomedicine, University of Basel, Basel, Switzerland

[3] Institute of Medical Bioinformatics and Systems Medicine (IBSM), Medical Center – University of Freiburg, Faculty of Medicine, University of Freiburg, Breisacher Strasse 153, 79110 Freiburg, Germany

[4] German Cancer Consortium (DKTK), Partner site University of Freiburg and German Cancer Research Center (DKFZ), Freiburg, Germany

[5] Signalling Research Centres BIOSS and CIBSS, University of Freiburg, Albertstrasse 19, 79104 Freiburg, Germany

[§] equal contribution

* To whom correspondence should be addressed:

Toma A. Yakulov

Renal Division

University Freiburg Medical Center – IMITATE

79106 Freiburg, Germany

E-mail: toma.antonov.yakulov@uniklinik-freiburg.de

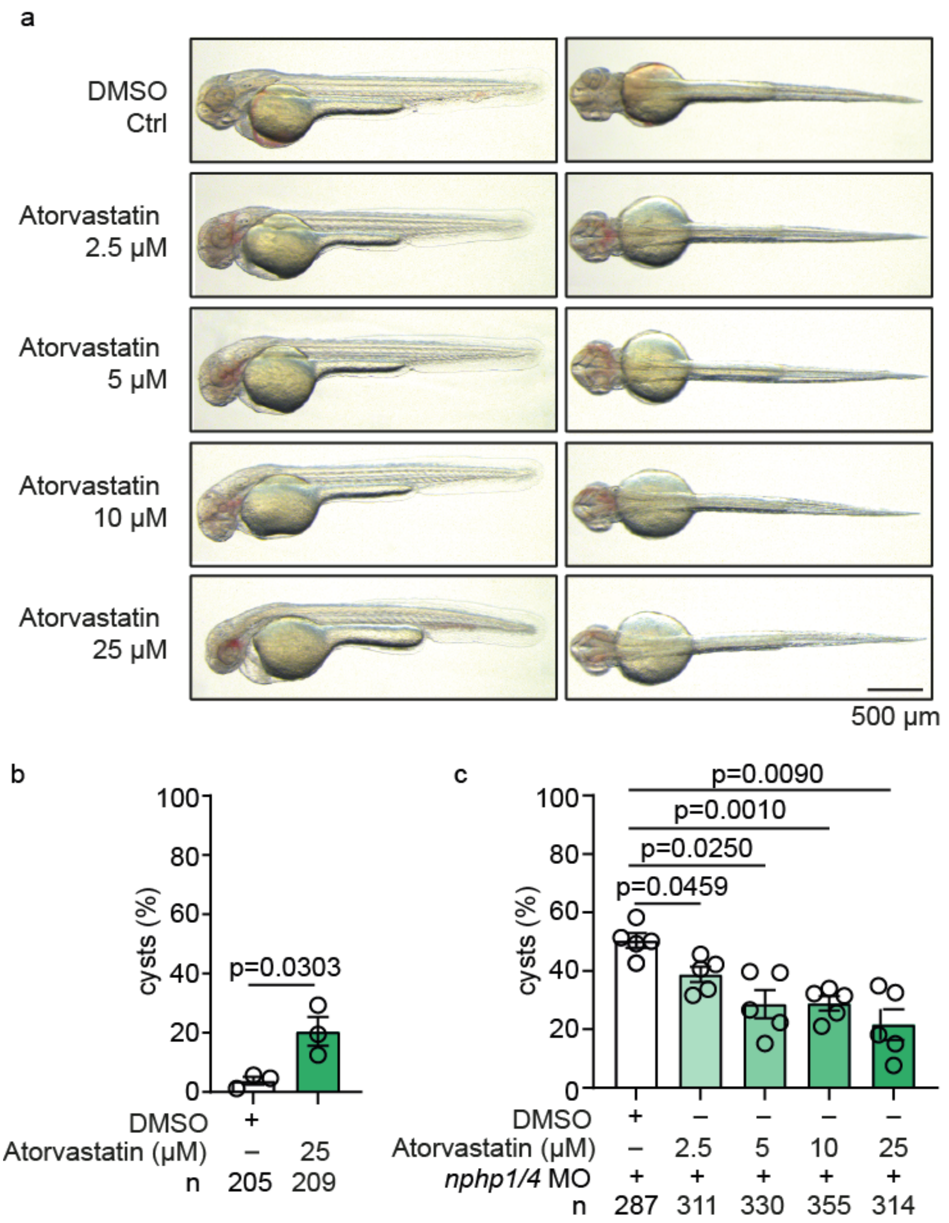


**Supplementary Figure S1 | Atorvastatin reduces cyst formation in zebrafish nephronophthisis model.** (**a**) Representative brightfield images of wild-type zebrafish embryos at 48 hpf following 24-hour treatment with DMSO (control) or increasing concentrations of Atorvastatin (2.5–25 µM). Embryos treated with Atorvastatin show no developmental toxicity or gross morphological abnormalities. Scale bar: 500 µm. (**b**) Quantification of cyst formation in wild-type *Tg(wt1b:GFP; cdh17:GFP)* embryos treated with DMSO or Atorvastatin (25 µM), demonstrating that Atorvastatin induces approximately 20% cyst formation (p = 0.0303). (**c**) Dose-response analysis reveals a concentration-dependent reduction in glomerular cysts in *nphp1/nphp4* morphant embryos treated with increasing concentrations of Atorvastatin (2.5–25 µM). Significant reductions in cyst formation are observed at concentrations of 2.5 µM (p = 0.0459), 5 µM (p = 0.0250), 10 µM (p = 0.0010), and 25 µM (p = 0.0090) compared to DMSO-treated morphants. Data are presented as mean ± SEM, and the total number of embryos analyzed per condition (n) is indicated below each graph.

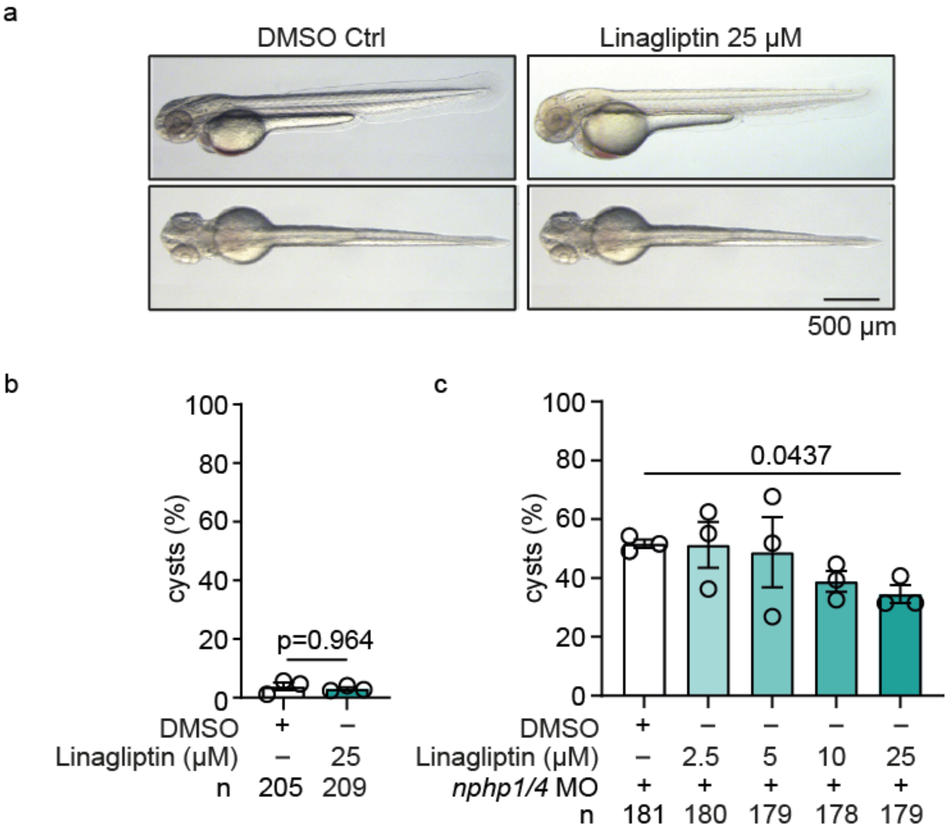


**Supplementary Figure S2 | Linagliptin reduces cyst formation in zebrafish nephronophthisis model.** (**a**) Representative brightfield images of wild-type zebrafish embryos at 48 hpf following 24-hour treatment with DMSO (control) or Linagliptin (25 µM). Embryos treated with Linagliptin show no developmental toxicity or morphological abnormalities. Scale bar: 500 µm. (**b**) Quantification of cyst formation in *Tg(wt1b:GFP; cdh17:GFP)* embryos treated with DMSO or Linagliptin (25 µM), demonstrating no cyst-inducing effects of Linagliptin. (**c**) Dose-response analysis reveals a concentration-dependent reduction in glomerular cysts in *nphp1/nphp4* morphant embryos treated with increasing concentrations of Linagliptin (2.5–25 µM). Significant reductions in cyst formation are observed at 25 µM ($p = 0.0437$) compared to DMSO-treated morphants. Data are presented as mean ± SEM, and the total number of embryos analyzed per condition (n) is indicated below each graph.

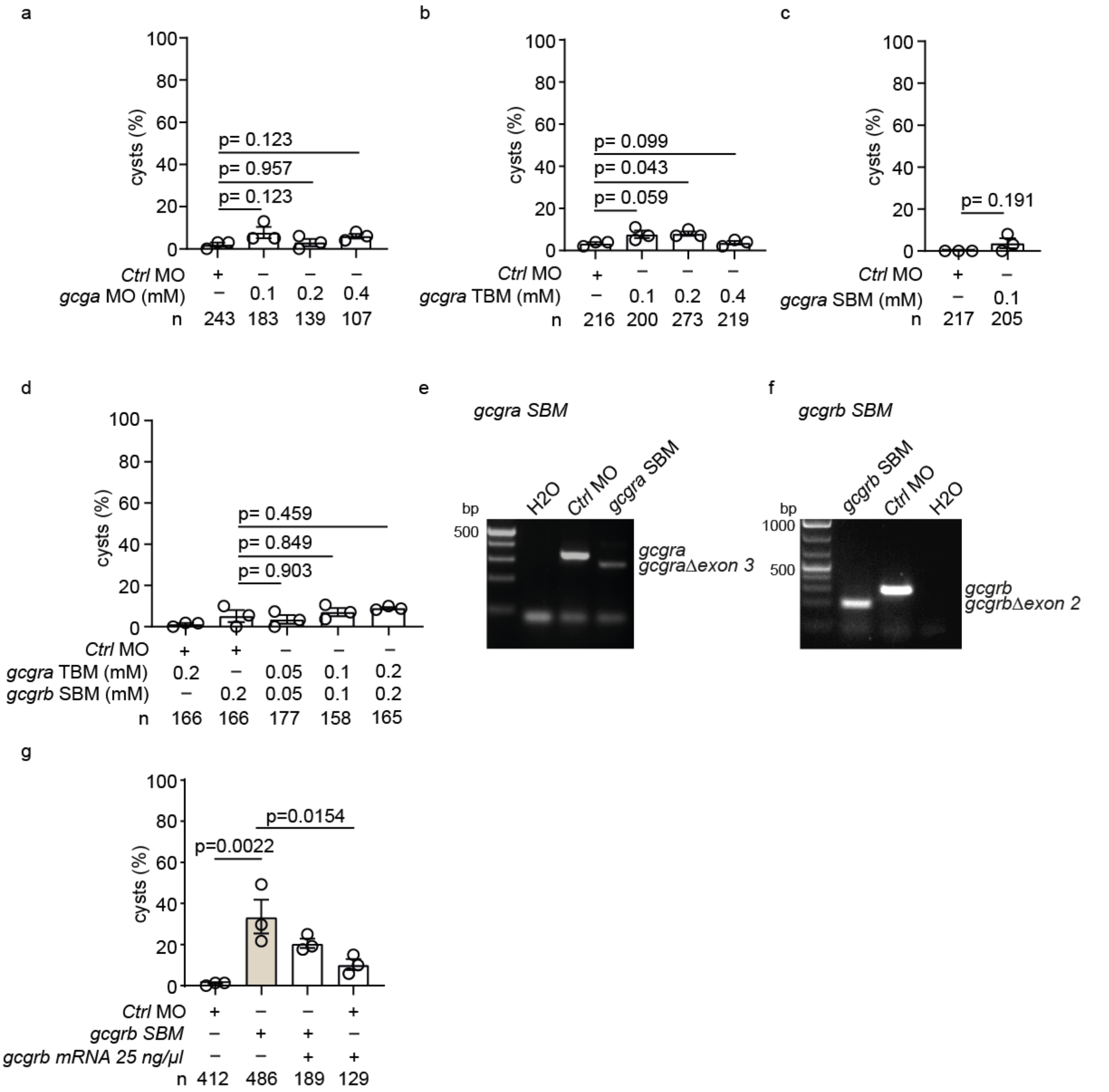


**Supplementary Figure S3 | Validation of *gcgra* and *gcgrb* splice-blocking morpholino efficacy, dose-response effects and rescue experiments.** (**a**) Quantification of cyst formation in wild-type embryos injected with increasing concentrations of gcga MO (0.1–0.4 mM). No significant cystogenesis is observed compared to Ctrl MO. (**b**) Dose-dependent quantification of cyst formation in wild-type embryos injected with *gcgra* TBM (0.1–0.4 mM), showing no significant effects on glomerular development compared to Ctrl MO. (**f**) Quantification of cyst formation in wild-type embryos injected with gcgra SBM (0.1 mM), showing no significant effects on glomerular development compared to *Ctrl* MO. (**d**) Quantification of cyst formation following combined knockdown of *gcgra* and *gcgrb*. Co-injection of suboptimal doses of *gcgra* TBM (0.05–0.2 mM) and *gcgrb* SBM (0.05–0.2 mM) does not result in cumulative effects on cystogenesis compared to individual knockdowns. (**e**) RT-PCR analysis demonstrates effective splice blocking by *gcgra* SBM, resulting in exon 3 deletion in the *gcgra* transcript. $H_2O$ serves as negative control, and *Ctrl* MO shows no splicing defects. (**f**) RT-PCR validation shows *gcgrb* SBM effectively induces exon 2 deletion in the *gcgrb* transcript compared to *Ctrl* MO. (**g**) Quantification of cyst formation in rescue experiments. Co-injection of *gcgrb* mRNA (25 ng/µl) reduces cyst formation in *gcgrb* SBM-depleted embryos in a concentration dependent manner. Data are presented as mean ± SEM. Each circle represents an independent experiment. The total number of embryos analyzed per condition (n) is indicated below each graph.

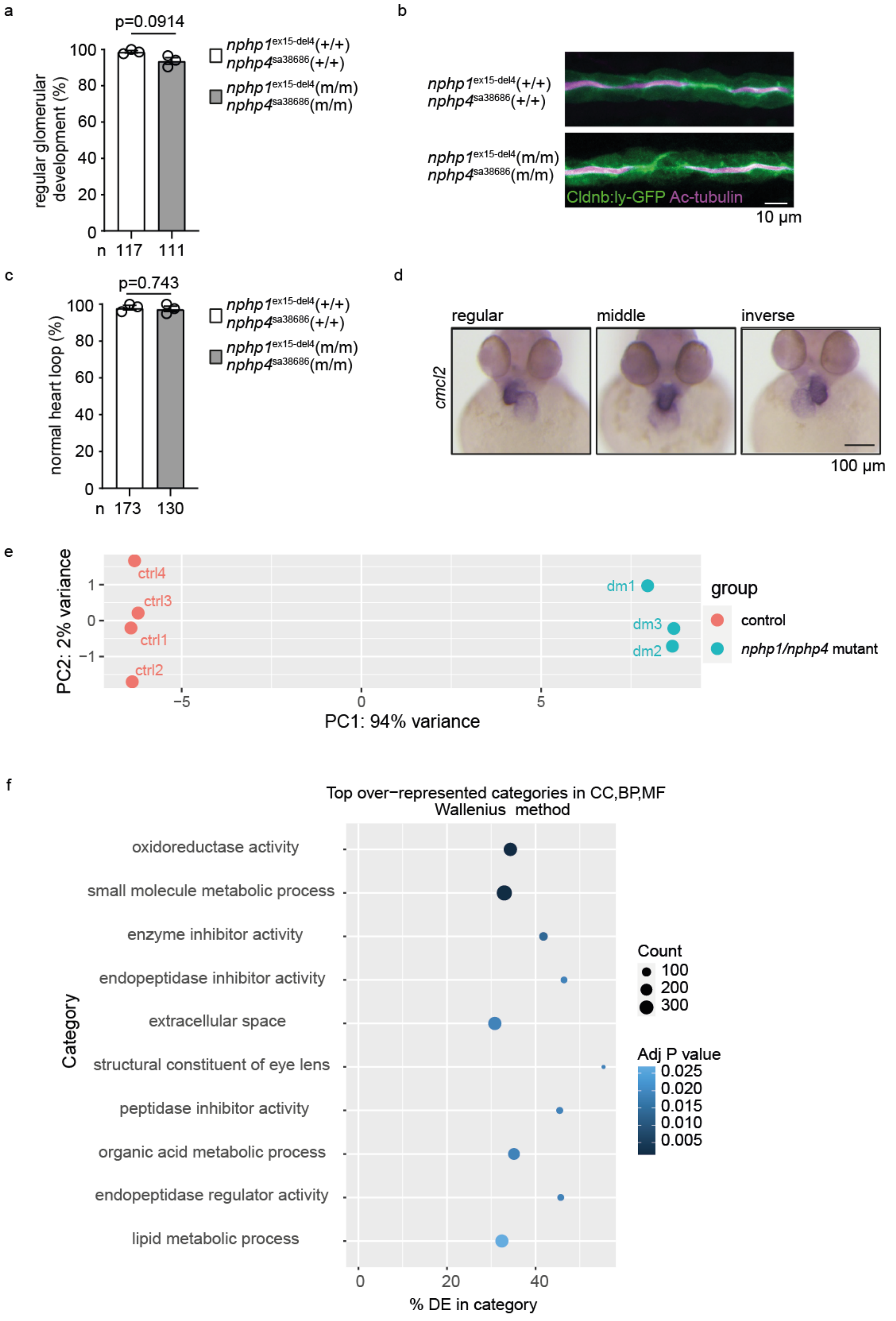


**Supplementary Figure S4 | *nphp1$^{ex15\text{-}del4}$/nphp4$^{sa38686}$* double mutants characterization and RNAseq analysis**. (**a**) Quantification of regular glomerular development in *nphp1$^{ex15\text{-}del}$; nphp4$^{sa38686}$* double mutant (m/m) embryos and wild-type siblings (+/+) at 48 hpf shows no significant difference.

(**b**) Representative confocal microscopy images of pronephric cilia in wild-type and *nphp1*$^{ex15\text{-}del4}$; *nphp4*$^{sa38686}$ double mutant embryos. *Tg(cldnb:ly-GFP)* (green) marks the pronephric tubules, and acetylated α-tubulin (magenta) labels cilia. Scale bar = 10 µm. (**c**) Quantification of normal heart looping in wild-type and double mutant embryos shows comparable development. (**d**) Representative in situ hybridization images showing *cmlc2* expression patterns categorized as regular, middle, or inverse heart looping. Scale bar = 100 µm. (**e**) Principal component analysis of RNA sequencing data demonstrates distinct transcriptional profiles between *nphp1*$^{ex15\text{-}del4}$; *nphp4*$^{sa38686}$ homozygous mutants (dm1–dm3, blue) and wild-type controls (ctrl1–ctrl4, red). PC1 accounts for 94% of the variance, while PC2 accounts for 2%. (**f**) Gene ontology enrichment analysis identifies overrepresented categories among differentially expressed genes in *nphp1*$^{ex15\text{-}del4}$; *nphp4*$^{sa38686}$ homozygous mutants compared to controls. Dot size represents the number of genes in each category, while color intensity indicates adjusted p-values. Data are presented as mean ± SEM. The total number of embryos analyzed per condition (n) is indicated below each graph.

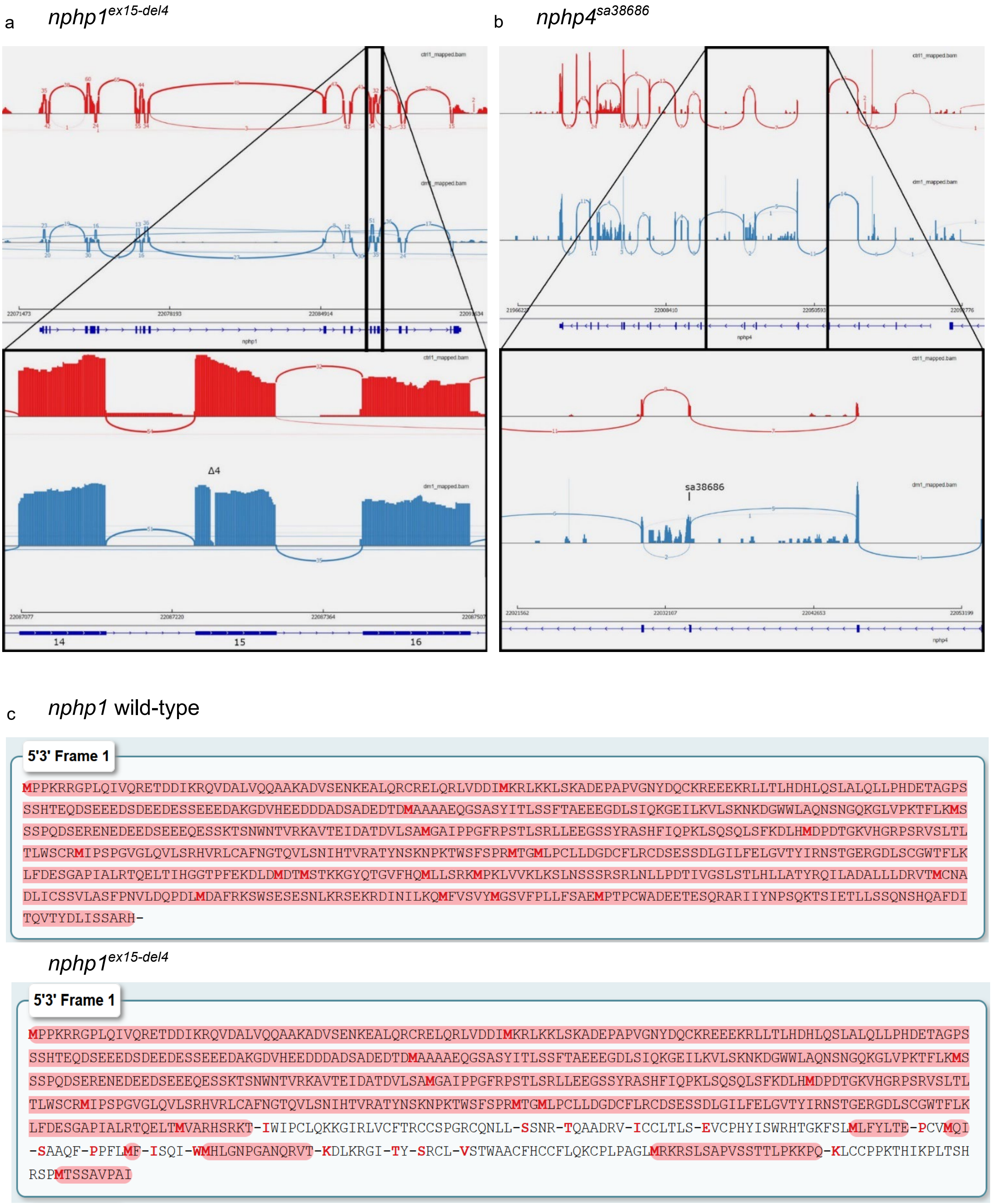


**Supplementary Figure S5 | Molecular characterization of *nphp1*$^{ex15\text{-}del4}$;*nphp4*$^{sa38686}$ mutant zebrafish lines.** (**a**) Schematic representation of the *nphp1* genomic locus showing the location of the 4-bp deletion in exon 15 (*nphp1*$^{ex15\text{-}del4}$). RNAseq reveals that the deletion results in a frameshift mutation and premature stop codon. Representative sequencing chromatograms confirm the wild-type sequence (top/red) and the 4-bp deletion in the mutant line (bottom/blue). (**b**) Schematic representation of the

*nphp4* genomic locus showing the splicing defect caused by the *sa38686* mutation. The mutation eliminates an essential splice site, resulting in aberrant splicing. (**c**) Predicted effect of the 4-bp deletion in *nphp1$^{ex15\text{-}del4}$* on the protein sequence. The frameshift caused by the deletion introduces a premature stop codon, resulting in a truncated protein. The wild-type protein sequence is shown alongside the altered sequence caused by the mutation.

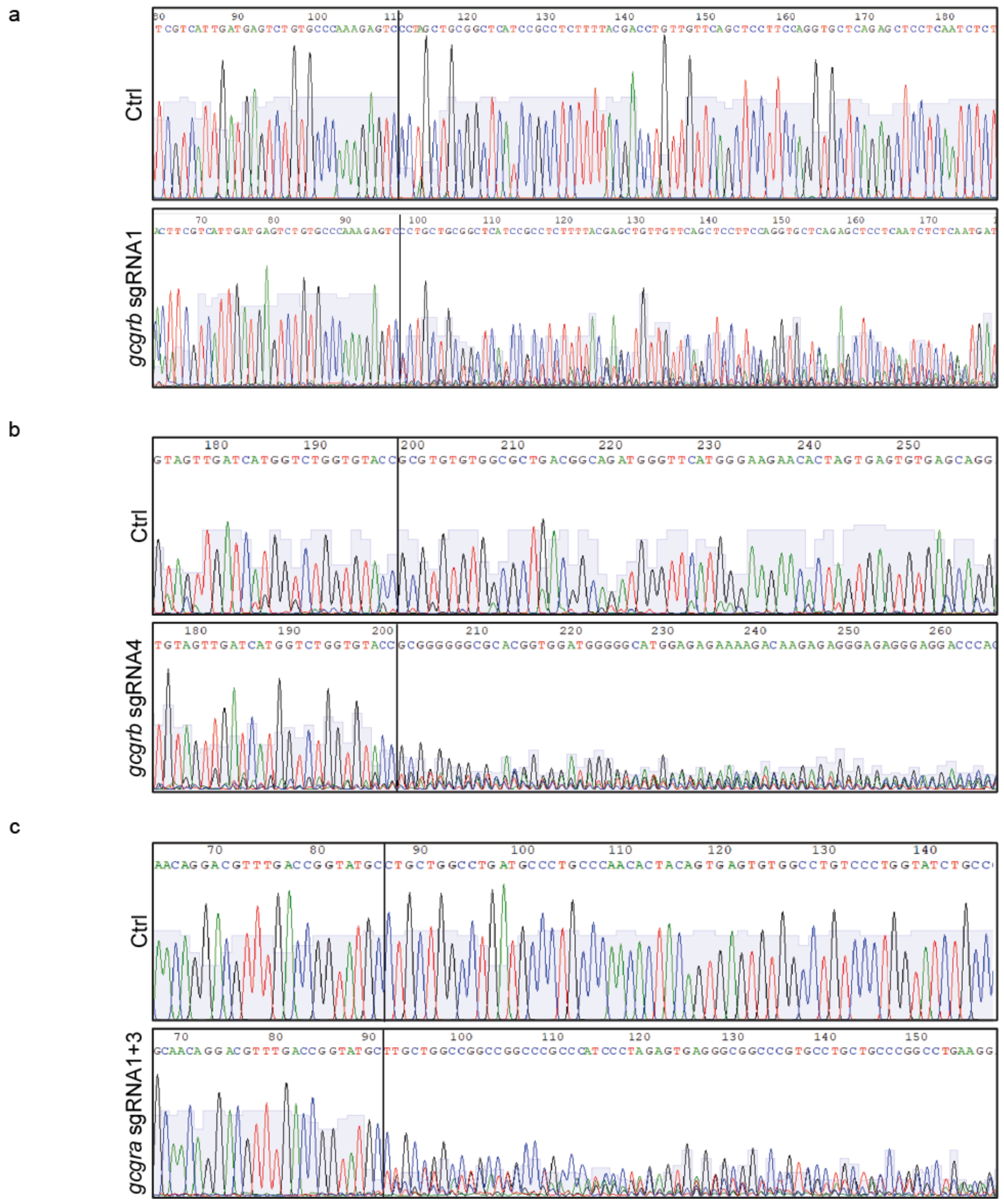


**Supplementary Figure S6 | CRISPR/Cas9-mediated targeting of *gcgrb* and *gcgra*, and validation of genome editing.** (**a**) Sanger sequencing chromatograms demonstrating successful genome editing in the *gcgrb* locus. Top panel shows wild-type control sequence, while bottom panel reveals mixed sequence peaks downstream of the sgRNA1 target site, indicating successful CRISPR/Cas9-mediated mutagenesis. (**b**) Validation of CRISPR/Cas9-mediated genome editing efficiency in the *gcgrb* locus. Top panel shows wild-type control sequence, while bottom panel demonstrates successful editing with mixed sequence peaks downstream of the sgRNA4 target site. (**b**) Validation of CRISPR/Cas9-mediated genome editing efficiency in the *gcgra* locus. Top panel shows wild-type control sequence, while bottom panel demonstrates successful editing with mixed sequence peaks downstream of the sgRNA1 and sgRNA3 target sites. The target sites are close to each other and cannot be distinguished on a chromatogram.

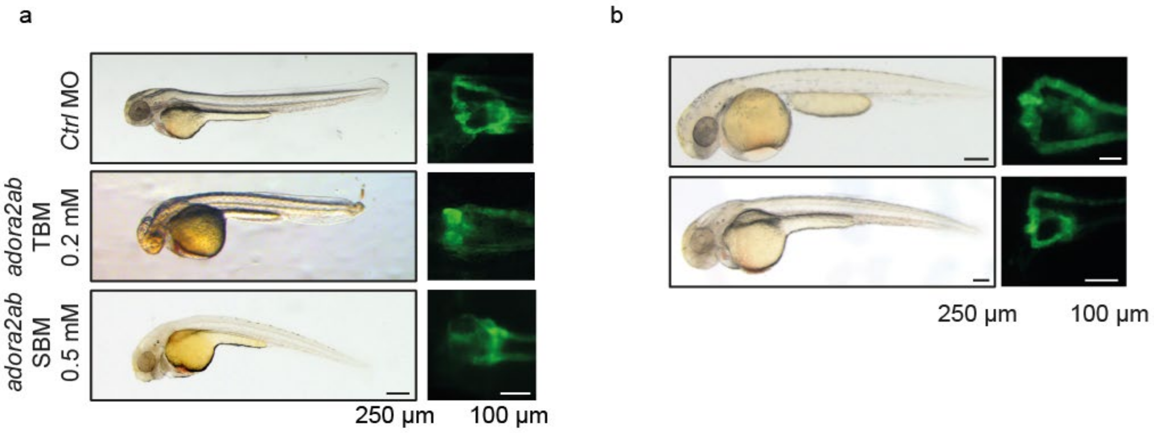


**Supplementary Figure S7 | *adora2ab* morpholino-induced phenotypes.** (**a**) Representative brightfield and fluorescence microscopy images showing morphological phenotypes of *adora2ab* MO-depleted emryos at 48 hpf. Bar = 250 µm (brightfield) and 100 µm (fluorescence). (**b**) Representative brightfield images (left panels) and fluorescence microscopy of pronephric cysts (right panels) at 48 hpf. Top panel: Control morpholino (0.2 mM) + *adora2ab* mRNA (30 ng/µl) showing normal development. Bottom panel: *adora2ab* TBM (0.2 mM) + *adora2ab* mRNA (30 ng/µl) demonstrating rescue of the cystic phenotype. Scale bars: brightfield = 250 µm, fluorescence = 100 µm.

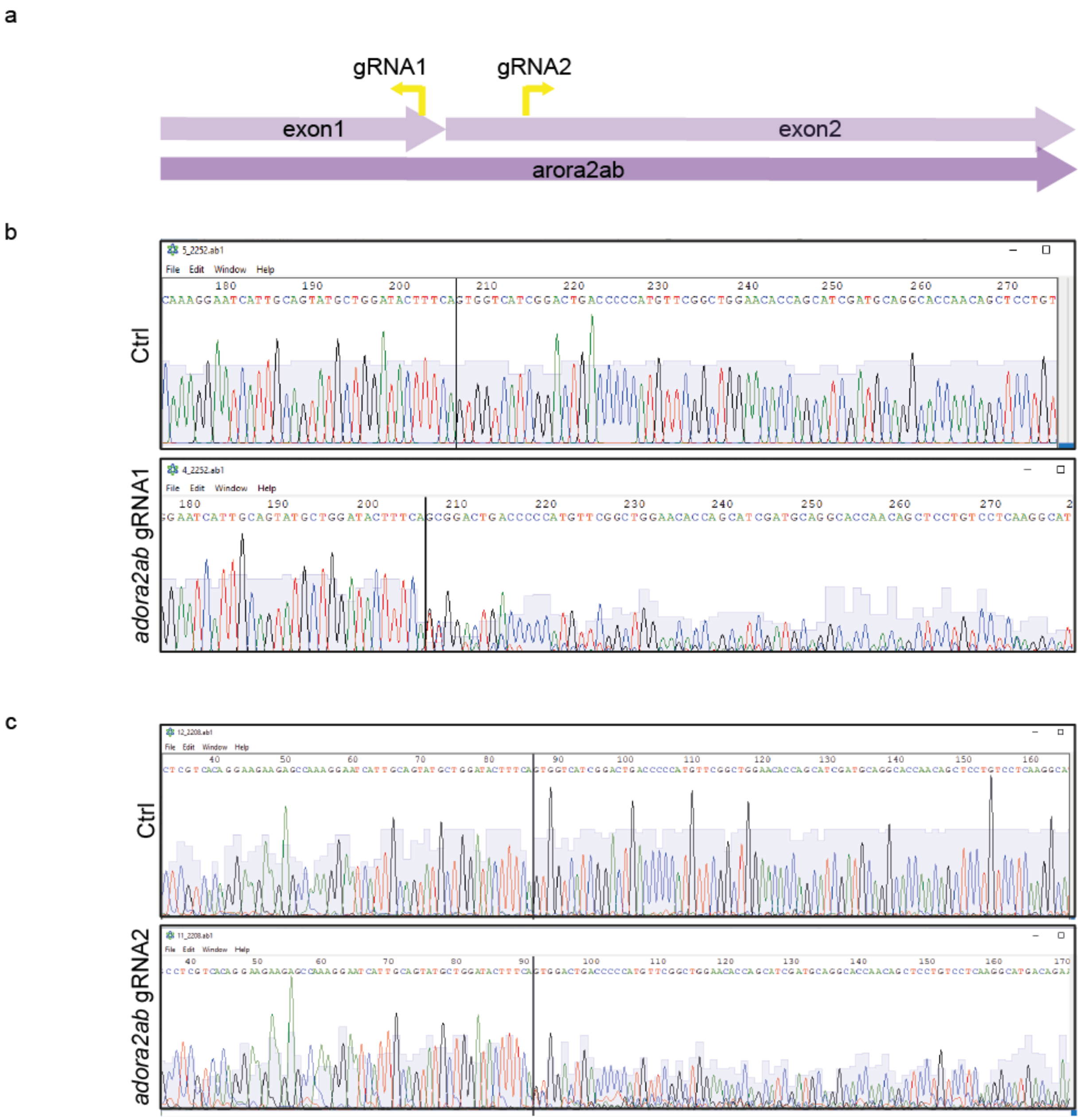


**Supplementary Figure S8 | CRISPR/Cas9-mediated targeting of *adora2ab* and validation of genome editing**. (**a**) Schematic representation of the *adora2ab* locus showing the targeting strategy with two small guide RNAs (gRNA1 and gRNA2) positioned at the exon1-exon2 boundary. (**b**) Sanger sequencing chromatograms demonstrating successful genome editing. Top panel shows wild-type control sequence, while bottom panel reveals mixed sequence peaks downstream of the gRNA1 target site, indicating successful CRISPR/Cas9-mediated mutagenesis. (**c**) Validation of genome editing efficiency using gRNA2. Top panel shows wild-type control sequence, while bottom panel demonstrates successful editing with mixed sequence peaks downstream of the gRNA2 target site.
