# Supplementary material for: Targeting GLP-1 Signaling Ameliorates Cystogenesis in a Zebrafish Model of Nephronophthisis
Source: Int J Mol Sci. 2025 Jul 30;26(15):7366. doi: 10.3390/ijms26157366 (PMC12347336; doi:10.3390/ijms26157366)
Supplement: Supplementary file 1 [file ijms-26-07366-s001.zip › Supplementary Methods.pdf]

Supplementary Methods

Targeting GLP-1 signaling ameliorates cystogenesis in a zebrafish model of nephronophthisis

Priska Eckert[1§], Maike Nöller[1§], Merle Müller[1§], Rebecca Haas[1§], Johannes Ruf[1§], Henriette Franz[2], Katharina Moos[3], Jia-ao Yu[1], Dongfang Zhao[1], Wanqiu Xie[1], Melanie Boerries[3,4], Gerd Walz[1,5], Toma A. Yakulov[1,*]

[1] Renal Division, University Freiburg Medical Center, Faculty of Medicine, University of Freiburg, Germany

[2] Department of Biomedicine, University of Basel, Basel, Switzerland

[3] Institute of Medical Bioinformatics and Systems Medicine (IBSM), Medical Center – University of Freiburg, Faculty of Medicine, University of Freiburg, Freiburg, Germany

[4] German Cancer Consortium (DKTK), Partner site University of Freiburg and German Cancer Research Center (DKFZ), Freiburg, Germany

[5] Signalling Research Centres BIOSS and CIBSS, University of Freiburg, Freiburg, Germany

[§] equal contribution

* To whom correspondence should be addressed:

Toma A. Yakulov

Renal Division

University Freiburg Medical Center – IMITATE

79106 Freiburg, Germany

E-mail: toma.antonov.yakulov@uniklinik-freiburg.de

ARRIVE compliance documentation

The ARRIVE essential 10

1. Study design

a. **Groups being compared**: Our study used several experimental comparisons:

- Wild-type embryos vs. *nphp1/nphp4* morphants
- DMSO-treated vs. drug-treated embryos (Omarigliptin, Linagliptin, Atorvastatin, Semaglutide,)
- *nphp1*$^{ex15\text{-}del4}$;*nphp4*^{sa38686} double mutants vs. wild-type siblings
- Control MO vs. gene-specific MO (*gcga*, *gcgra*, *gcgrb*, *adora2ab*)
- Normal gene expression vs. CRISPR/Cas9-mediated gene targeting
- Suboptimal-dose MO combinations to identify genetic interactions

b. **Experimental unit**: Individual zebrafish embryos served as the experimental unit in all analyses.

2. Sample size

Sample sizes were determined based on previous experience with similar zebrafish experiments. For the drug screen, groups of 20-25 embryos in triplicate were used to ensure statistical power while maintaining feasibility. For validation experiments, the exact number of embryos analyzed per condition (n) is indicated below each graph in the figures. For RNA sequencing, 15-50 embryos at 48 hpf were pooled for each sample, with 4 biological replicates per condition.

3. Inclusion and exclusion criteria

For drug screen evaluation, three specific criteria were established:

1. Individual dishes were only counted if 10 or more embryos survived the treatment
2. Triplicates were only counted if all 3 dishes had sufficient surviving embryos
3. The whole experiment was only considered if the amount of cysts in the DMSO treatment was between 25% and 75%

Embryos with developmental abnormalities unrelated to the experimental variables (e.g., severe malformations) were excluded from analysis. Embryos that died during experimental procedures were also excluded. For differential expression analysis of RNA sequencing data of Omarigliptin vs. DMSO-treated embryos, two samples (DMSO_1 and Omarigliptin_2) were excluded due to observed clustering differences in PCA analysis, indicating potential developmental stage differences. Similarly, for differential expression analysis of RNA sequencing data of *nphp1*$^{ex15\text{-}del4}$;*nphp4*^{sa38686} double mutants vs. wild-type siblings, one sample (dm4) was excluded due to observed clustering differences in PCA analysis.

4. Randomization

For all experiments, embryos were randomly allocated to experimental groups. After collection, fertilized eggs were pooled and then randomly distributed to experimental conditions. For drug treatments, embryos were first injected with MO and then randomly assigned to drug or control treatments. To minimize potential confounders, experiments were designed with standardized protocols across all treatment groups. Drug treatments were performed in multi-well plates with embryos randomly distributed to minimize positional effects. Environmental factors including temperature, light cycles, and water quality were strictly controlled and maintained consistently across all experimental groups. All experiments included appropriate controls processed simultaneously with experimental groups to account for batch effects.

5. Blinding

The investigators were not blinded during group allocation but were blinded during outcome assessment for phenotypic analyses whenever possible.

6. Outcome measures

Primary outcome measures included:

- Percentage of embryos with pronephric glomerular cysts at 48 hpf, assessed by fluorescence microscopy
- Ciliary morphology assessed by acetylated tubulin immunostaining and confocal microscopy
- Gene expression changes measured by RNA sequencing and RT-PCR
- Heart looping patterns assessed by whole-mount in situ hybridization

All outcomes were predefined before starting the experiments.

7. Statistical methods

Statistical significance for multiple comparisons was determined using Dunnet's test, which compares each experimental group to a control group while controlling for family-wise error rate. For single comparisons, Student's t-test was used, with $p < 0.05$ considered statistically significant. Data are presented as mean ± SEM (standard error of the mean). Each circle in graphs represents an independent experiment. For RNA sequencing data, gene count normalization and differential gene expression analysis were performed as described in the main methods part.

8. Experimental animals

Species: Zebrafish (Danio rerio)
Strain background: Standard TL strain for all transgenic and mutant lines
Developmental stage: Embryos were analyzed up to 48 hpf

Sex: Sex is not determined at these early developmental stages
Transgenic lines:

- *Tg(cdh17:GFP;wt1b:GFP)* for visualization of pronephric structures
- *Tg(-8.0 cldnb:Ly-GFP;wt1b:GFP)* for visualization of pronephric structures

Mutant lines:

- $nphp1^{ex15\text{-}del4};nphp4^{sa38686}$: 4-bp deletion in exon 15 of nphp1 resulting in frameshift mutation and premature stop codon, and splice site mutation that eliminates an essential splice site, resulting in aberrant splicing

9. Experimental procedures

See main text.

10. Results

For each analysis, the exact value of n in each experimental group is indicated in the figure legends. No data points were excluded from analyses except as specified in the Inclusion/Exclusion Criteria section. All attempts at replication were successful, and the results reported include data from at least three independent experiments for most analyses. Complete numerical data for all experiments are available upon request.

Housing and husbandry

Zebrafish were maintained in a recirculating system with a 12-hour light/12-hour dark cycle at 28 °C. Water quality parameters (pH, conductivity, temperature) were monitored daily and maintained within optimal ranges. Adult fish were fed twice daily with a combination of dry flake food and live Artemia. Adult density was maintained at no more than 7 fish per liter in appropriately sized tanks.

Ethical statement

All animal procedures were performed in accordance with the guidelines of the German animal protection law and approved by the local animal ethics committee at the Regierungspräsidium Freiburg (permit G-21/146). Every effort was made to minimize the number of animals used and their suffering.

Animal care and monitoring

Embryos were monitored daily for signs of distress or developmental abnormalities. Any embryos showing signs of infection or severe developmental abnormalities unrelated to experimental manipulations were humanely euthanized. As the experiments were conducted on

embryos younger than 5 days post-fertilization (before independent feeding begins), procedures fall under regulations with reduced severity classifications.
